# Proteomic analysis revealed the roles of *YRR1* deletion in enhancing the vanillin resistance of *Saccharomyces cerevisiae*

**DOI:** 10.1186/s12934-021-01633-z

**Published:** 2021-07-23

**Authors:** Wenyan Cao, Weiquan Zhao, Bolun Yang, Xinning Wang, Yu Shen, Tiandi Wei, Wensheng Qin, Zailu Li, Xiaoming Bao

**Affiliations:** 1 State Key Laboratory of Biobased Material and Green Papermaking, School of Bioengineering, Key Laboratory of Shandong Microbial Engineering, Qilu University of Technology (Shandong Academy of Sciences), 3501 Daxue Road, Jinan, 250353 China; 2 State Key Laboratory of Microbial Technology, Institute of Microbial Technology, Shandong University, Binhai Road 72, Qingdao, 266237 China; 3 Department of Biology, Lakehead University, 955 Oliver Rd, Thunder Bay, ON P7B 5E1 Canada

**Keywords:** *YRR1*, Quantitative proteomics, Vanillin resistance, Bioethanol, *Saccharomyces cerevisiae*

## Abstract

**Background:**

Vanillin is one of the important phenolic inhibitors in *Saccharomyces cerevisiae* for bioconversion of lignocellulosic materials and has been reported to inhibit the translation process in cells. In our previous studies, it was confirmed that the deletion of the transcription factor gene *YRR1* enhanced vanillin resistance by promoting some translation-related processes at the transcription level. In this work, we investigated the effects of proteomic changes upon induction of vanillin stress and deletion of *YRR1* to provide unique perspectives from a transcriptome analysis for comprehending the mechanisms of *YRR1* deletion in the protective response of yeast to vanillin.

**Results:**

In wild-type cells, vanillin reduced two dozens of ribosomal proteins contents while upregulated proteins involved in glycolysis, oxidative phosphorylation, and the pentose phosphate pathway in cells. The ratios of NADPH/NADP^+^ and NADH/NAD^+^ were increased when cells responded to vanillin stress. The differentially expressed proteins perturbed by *YRR1* deletion were much more abundant than and showed no overlaps with transcriptome changes, indicating that Yrr1 affects the synthesis of certain proteins. Forty-eight of 112 upregulated proteins were involved in the stress response, translational and transcriptional regulation. *YRR1* deletion increased the expression of *HAA1-*encoding transcriptional activator, *TMA17-*encoding proteasome assembly chaperone and *MBF1*-encoding coactivator at the protein level, as confirmed by ELISA. Cultivation data showed that the overexpression of *HAA1* and *TMA17* enhanced resistance to vanillin in *S. cerevisiae*.

**Conclusions:**

Cells conserve energy by decreasing the content of ribosomal proteins, producing more energy and NAD(P)H for survival in response to vanillin stress. Yrr1 improved vanillin resistance by increasing the protein quantities of Haa1, Tma17 and Mbf1. These results showed the response of *S. cerevisiae* to vanillin and how *YRR1* deletion increases vanillin resistance at the protein level. These findings may advance our knowledge of how *YRR1* deletion protects yeast from vanillin stress and offer novel targets for genetic engineering of designing inhibitor-resistant ethanologenic yeast strains.

**Supplementary Information:**

The online version contains supplementary material available at 10.1186/s12934-021-01633-z.

## Background

*Saccharomyces cerevisiae* is a traditionally competitive cell factory for bioethanol and the production of other desired chemicals due to its superior robustness and the ease in which genetic manipulation can be performed [1, 2]. Lignocellulosic materials have been viewed as a major resources for bioethanol production worldwide because they are available in abundance, are renewable, and do not compete with food resources [3]. However, the pretreatment of lignocellulose is inevitably generates many inhibitors, such as organic acids, furans, and phenolic compounds, which hamper microorganism growth and fermentation [4]. Vanllin, a guaiacyl phenol, has been considered as an important inhibitor of lignocellulosic hydrolysates, as it inhibits the viability of many microorganisms at very low concentration [5]. The concentrations of vanillin ranges from 1 to 26 mM according to the types of biomass materials and the method of pretreatment [6, 7]. In addition, as one of the most widely useful flavoring agents in the food and cosmetics industry, vanillin is produced by microorganisms from glucose or ferulic acid, which is other components of lignin [8–11]. Thus, it is essential to understand the physiological mechanism of how vanillin inhibits *S. cerevisiae* growth and fermentation during the generation of vanillin-resistant *S. cerevisiae* cell factories to improve bioethanol and vanillin production.

In our previous work, we reported that the deletion of the multidrug resistance-related transcription factor gene *YRR1* remarkably enhanced *S. cerevisiae* vanillin resistance. The rate of growth and vanillin transformation to vanillyl alcohol in the *YRR1*-deleted strain under vanillin stress was faster than that in the wild-type yeast. This phenomenon is generally evident in different background strains (BY4741 and CEN.PK2-1C) [12]. Transcriptome analysis showed that the genes related to ribosome biogenesis and rRNA processing are upregulated in the *YRR1*-deleted strain compared to the wild-type strain under vanillin stress [12]. Among the upregulated genes, the overexpression of *DBP2*, encoding RNA helicase and participating in ribosome biogenesis, was confirmed to enhance vanillin resistance [12]. Ribosomes and rRNA are vital for translation and protein synthesis [12]. Thus, *YRR1* was thought to impact translation and protein synthesis. In addition, Iwaki et al. [13] have revealed that vanillin represses translational initiation by blocking polysome assembly, which led to the accumulation of cytoplasmic processing bodies (P-bodies) and stress granules (SGs). Furfural, 5-hydroxymethyl furfural (HMF), and acidic stress also exhibited the same phenomenon [14, 15].

Therefore, global mRNA profiles alone are not sufficient to represent the changes in the biological system because translational and post-translational regulation mechanisms, protein degradation, and other processes have not been taken into account. Transcript levels frequently do mismatch the corresponding protein levels [16, 17].

In general, to gain a complete insight into how *YRR1* deletion protects yeast from vanillin stress, in this study, we conducted a quantitative proteomic analysis of yeast treated with specific vanillin stress and deletion of *YRR1*, respectively. The proteomic data were analyzed in combination with previous transcriptome data obtained under the same conditions. We found that *YRR1* deletion increased Haa1, Tma17 and Mbf1 expression at the protein level, and overexpression of *HAA1* and *TMA17* enhanced the resistance of yeast to vanillin. This work enriches the knowledge of the protective response conferred by *YRR1* deletion to vanillin stress and offered novel strategies for genetic engineering manipulation to improve microbe resistance.

## Results and discussion

### Quantitative proteomic analysis reveals remarkable alterations in the protein abundance in response to vanillin

**Fig. 1:**
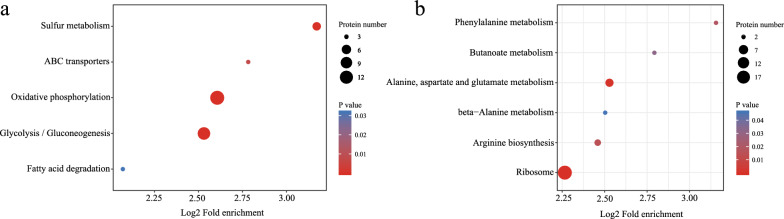
Statistics of KEGG pathway enrichment of DEPs of BY4741 response to 5 mM vanillin. BY4741 cultivated with 5 mM vanillin for 15 h or without vanillin for 6 h when OD_600_ reached to 1.6. **a** Upregulated proteins; **b** down-regulated proteins. The log_2_ fold enrichment is the log_2_ converted value of the ratio of DEP number annotated in this pathway term to all protein numbers annotated in this pathway term. Greater log_2_ fold enrichment indicates a greater effect of vanillin on the analyzed pathway

To investigate the changes in protein abundance perturbed by vanillin stress, the proteomic differences were examined with the laboratory strain BY4741 cultivated with 5 mM vanillin for 15 h or without vanillin for 6 h when OD_600_ reached to 1.6. We used 1.2-, 1.3-, 1.5- and 2-fold thresholds to analyze the differentially expressed proteins (DEPs). The numbers of 1.2-, 1.3-, 1.5- and 2-fold DEPs was 1002, 218, 85 and 19, respectively. In addition, the corresponding numbers of DEPs between strains BY4741 and BY4741(*yrr1Δ*) without vanillin treatment was 198, 121, 32 and 0. According to the advice offered by Jingjie PTM Biolab, a proteomics company, and many published articles on proteomics [18, 19], we chose 1.3 as the threshold. According to our stringent data-mining criteria, 135 proteins were upregulated and 83 proteins were downregulated in response to vanillin (*P*-value < 0.05). Functional annotation of all the identified proteins was conducted based on the Gene Ontology (GO) database [20] and the Kyoto Encyclopedia of Genes and Genomes (KEGG) database [21]. The KEGG pathway enrichment of DEPs suggests that the upregulated protein genes are mainly involved in the functional categories of oxidative phosphorylation and glycolysis (Fig. 1a). In contrast, downregulated protein genes are largely involved with the function of ribosome (Fig. 1b).

### Proteomes exhibited visible differences in the presence of vanillin according to the transcriptome analysis

**Fig. 2:**
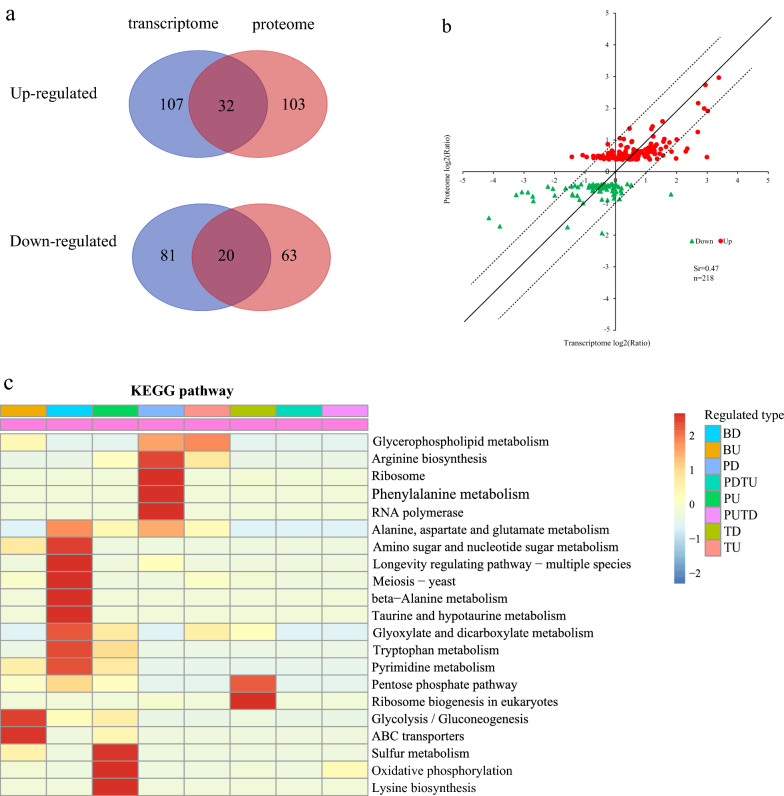
Proteomic analysis integrated with transcriptomics data of BY4741 under 5 mM vanillin stress. BY4741 cultivated with 5 mM vanillin for 15 h or without vanillin for 6 h when OD_600_ reached to 1.6. **a** The Venn diagram shows the overlaps of the transcriptomes and proteomes; **b** a scatter plot of differently expressed protein (n = 218) ratios versus the corresponding mRNA expression ratios. The plotted x = y line indicates data points showing a perfect correlation between mRNA and proteins. Dashed lines indicate a twofold deviation between the mRNA and protein expression ratios. The greater the absolute value of *Sr* (*Spearman*), the stronger the correlation; **c** the heatmap of KEGG pathway cluster analysis of differently expressed genes/proteins: BD and BU (both of protein and mRNA are down-regulated or upregulated); PU and PD (only upregulated or down-regulated at protein level); TU and TD (only upregulated or down-regulated at the mRNA level); PDTU (down-regulated at protein level and upregulated at mRNA level); PUTD (upregulated at protein level and down-regulated at mRNA level); red indicates strong enrichment, and blue indicates weak enrichment

Considering of the repression of the translation initiated by vanillin, mRNA most likely cannot represent protein expression. Thus, a proteomic dataset was compared to data obtained from a previous transcriptomic outcome (GEO accession number: GSE89854) to investigate the correlation between mRNA and protein levels [12]. Overlaps (i.e., homodirectional) between DEPs and differentially expressed genes (DEGs) are shown in the Venn diagrams (Fig. 2a). Only 23.8% of these DEPs exhibited a homodirectional change at the level of their transcript counterparts. A scatter plot was used to compare high-quality protein with the transcript expression ratios for all 218 DEPs (Fig. 2b). The positive Spearman rank correlation coefficient (Sr) of 0.47 that was calculated for these 218 DEPs revealed a weak correlation between the transcriptome and proteome. This poor correlation between changes in protein and the corresponding transcripts indicated that the changes in protein expression in response to vanillin stress are mainly regulated at the posttranscriptional level.

The DEPs and DEGs enriched (*P* < 0.05) in KEGG pathways are listed in Fig. 2c. There were total nineteen glycolysis-related genes and ten ABC transporters in S288C *S. cerevisiae* according to Saccharomyces Genome Database (SGD). At both the mRNA and protein levels, four proteins (Tdh1, Tdh2, Hxk2 and Eno1) in glycolysis-related pathways and three ABC transporters (Pdr5, Snq2 and Pdr12) were upregulated, which indicates that vanillin regulates the expression of these genes at the transcriptional level. On the other hand, at the protein level, the ribosomal proteins and RNA polymerases were downregulated significantly, which indicated that vanillin inhibited the translation of proteins in these two pathways. However, this inhibition was gene-specific. Nguyen et al. [22] found that both of *ADH6* and *ADH7*, which encode medium-chain alcohol dehydrogenases, were transcriptionally upregulated by vanillin stress. However, only the protein of *ADH7* was increased, while the protein of *ADH6* decreased under vanillin stress. The mechanism behind this finding is not clear, but it is probably related to the transcriptome and proteome inconsistency.

### Ribosomal proteins and rRNA processing-related proteins were significantly decreased in response to vanillin stress

Although the transcriptome showed that 13 genes related to ribosome biogenesis were notably downregulated (Table 1), no changes were exhibited at the corresponding protein level in response to vanillin stress [12]. However, twenty ribosomal proteins showed decreased expression under vanillin stress, including 5 small ribosomal subunits (40S) and 15 large ribosomal subunits (60S) (Table 1). Moreover, one ribosome biogenesis-related protein (Rlp24) [23], three proteins involved in rRNA processing (Esf1, Nsa2, Pop8), two RNA polymerase I subunits (Rpa12 and Rpa190), one RNA polymerase III subunit (Rpc11), tRNA 4-demethylwyosine synthase Tyw1, and an ATPase for tRNA processing of Nbp35 also displayed a visible decrease in protein abundance. These downregulated proteins are constituents of the cellular translational machine, which indicates that vanillin blocks translation efficiency and protein synthesis by reducing the quantity of ribosomal proteins available. This finding coincides with the fact that vanillin inhibits translation initiation, resulting in cells not needing many more ribosomes.

**Table 1:** Differentially expressed genes/proteins related to ribosomal proteins, ribosome biogenesis and rRNA processing induced by 5 mM vanillin stress in BY4741

ORF	Protein/gene	Description	DEPs ratio^a^/DEGs log_2_ ratio^b^
Ribosomal proteins at protein level			DEPs ratio
* YDR025W*	Rps11a	Ribosomal 40S subunit protein S11A	0.724
* YPR132W*	Rps23b	Ribosomal 40S subunit protein S23B	0.753
* YLR388W*	Rps29a	Ribosomal 40S subunit protein S29A	0.660
* YDL061C*	Rps29b	Ribosomal 40S subunit protein S29B	0.610
* YNL096C*	Rps7b	Ribosomal 40S subunit protein S7B	0.763
* YKL006W*	Rpl14a	Ribosomal 60S subunit protein L14A	0.695
* YHL001W*	Rpl14b	Ribosomal 60S subunit protein L14B	0.709
* YGR148C*	Rpl24b	Ribosomal 60S subunit protein L24B	0.727
* YBL092W*	Rpl32	Ribosomal 60S subunit protein L32	0.755
* YPL143W*	Rpl33a	Ribosomal 60S subunit protein L33A	0.656
* YOR234C*	Rpl33b	Ribosomal 60S subunit protein L33B	0.644
* YIL052C*	Rpl34b	Ribosomal 60S subunit protein L34B	0.707
* YMR194W*	Rpl36a	Ribosomal 60S subunit protein L36A	0.752
* YPL249C-A*	Rpl36b	Ribosomal 60S subunit protein L36B	0.743
* YLR185W*	Rpl37a	Ribosomal 60S subunit protein L37A	0.740
* YLR325C*	Rpl38	Ribosomal 60S subunit protein L38	0.719
* YNL162W*	Rpl42a	Ribosomal 60S subunit protein L42A	0.765
* YPR043W*	Rpl43a	Ribosomal 60S subunit protein L43A	0.700
* YPL198W*	Rpl7b	Ribosomal 60S subunit protein L7B	0.683
* YHL033C*	Rpl8a	Ribosomal 60S subunit protein L8A	0.746
rRNA processing at protein level			
* YDR365C*	Esf1	Nucleolar protein involved in pre-rRNA processing	0.742
* YBL018C*	Pop8	Ribonuclease P	0.750
* YER126C*	Nsa2	rRNA-processing protein NSA2	0.727
* YDR279W*	Rnh202	Ribonuclease H2 subunit	0.710
* YJR063W*	Rpa12	RNA polymerase I core subunit RPA12	0.750
* YOR341W*	Rpa190	RNA polymerase I core subunit RPA190	0.746
* YDR045C*	Rpc11	RNA polymerase III core subunit RPC11	0.730
* YLR009W*	Rlp24	Ribosome biosynthesis protein	0.719
Ribosome biogenesis at mRNA level			DEGs log_2_ ratio
* YOL077C*	Brx1	Ribosome biogenesis protein	− 1.4
* YAL025C*	Mak16	Ribosome biosynthesis protein	− 1.2
* YOL041C*	Nop12	Involved in biogenesis of large 60S ribosomal subunit	− 1.3
* YPL043W*	Nop4	mRNA-binding ribosome biosynthesis protein	− 1.0
* YDR496C*	Puf6	Negative regulation of translation, ribosomal large subunit biogenesis	− 1.5
* YHR066W*	Ssf1	rRNA-binding ribosome biosynthesis protein	− 1.1
* YPL226W*	New1	ATP binding cassette protein	− 1.1
* YGR159C*	Nsr1	Required for pre-rRNA processing and ribosome biogenesis	− 1.4
* YDR398W*	Utp5	Subunit of U3-containing small subunit processome complex	− 1.0
* YDR299W*	Bfr2	rRNA-processing protein	− 1.3
* YNL112W*	Dbp2	DEAD-box ATP-dependent RNA helicase	− 1.5
*YAL025C*	Mak16	Ribosome biosynthesis protein	− 1.2
* YOL041C*	Nop12	Nucleolar protein involved in pre-25S rRNA processing	− 1.3

BY4741 cultivated with 5 mM vanillin for 15 h or without vanillin for 6 h when OD_600_ reached to 1.6

^a^DEPs ratio (BY4741with/without 5 mM vanillin stress) which is less than 1 means down-regulation at protein level; *P*-value ≤ 0.05

^b^log2 ratio (BY4741with/without 5 mM vanillin stress) which is less than 0 means down-regulation at mRNA level. False discovery rate (FDR) ≤ 0.001

Under same conditions, a proteomic analysis were performed with another haploid laboratory strain CEN.PK2-1C, which has a different genetic background than BY4741. After treatment of CEN.PK2-1C with or without vanillin, the proteome data showed that vanillin treatment decreased the content abundance of certain proteins related to ribosomes, as indicated by a pathway enrichment analyses (Additional file 1: Fig. S1). Therefore, these results demonstrated that the reduction in ribosome biogenesis is likely representative of the cellular conservation of energy needed for survival under vanillin stress, since ribosome biogenesis demands a great supply of energy [24, 25].

### Vanillin stress increased the quantity of proteins involved in energy generation

As shown in Table 2, a number of enzymes involved in energy generation pathways, such as glycolysis, oxidative phosphorylation, and the pentose phosphate pathway (PPP), were dramatically upregulated. Six glycolytic enzymes (Pyc1, Tdh1, Tdh2, Hxk2, Eno1, and Gpm2) associated with glycolysis were remarkably increased. In particular, Hxk2, the predominant enzyme that catalyzes the first irreversible step of glycolysis [26], was upregulated more than 1.5-fold. Hxk2 is one of two rate-limiting enzymes in glycolysis [27]. Moreover, the proteins related to adenosine triphosphatase (ATP) synthesis, including the five ATP synthase subunits (Atp20, Atp7, Atp15, Atp14, and Tim11), three cytochrome c oxidase subunits (Cox4, Cox12, and Cox8), four ubiquinol cytochrome c reductase subunits (Qcr9, Qcr2, Cor1, and Qcr6), and one ATPase (Pma2), were also increased significantly. Upregulated glycolysis and oxidative phosphorylation pathways tend to supply more ATP for cellular survival as the detoxification of vanillin consumes energy. For example, when facing vanillin stress, some ATP binding cassette (ABC) transporters are upregulated, such as Pdr5 and Snq2 (upregulated 1.68 and 3.78 times, respectively, at the protein level), to extrude vanillin from the cells, which requires a large amount of energy. These two transporters were upregulated at the mRNA level, and their overexpression was confirmed to shorten the lag phase under vanillin stress [12]. A similar result was also found in the proteome of CEN.PK2-1C treated with vanillin, in which a visible amount of upregulated protein was related to glycolysis (Additional file 1: Fig. S2).

**Table 2:** Differentially expressed proteins related to energy metabolism induced by 5 mM vanillin stress in BY4741

ORF	Protein name	Description	DEPs ratio^a^
ATP biosynthetic process			
* YPR020W*	Atp20	F1F0 ATP synthase subunit g	1.37
* YKL016C*	Atp7	F1F0 ATP synthase subunit d	1.33
* YPL271W*	Atp15	F1F0 ATP synthase subunit epsilon	1.43
* YLR295C*	Atp14	F1F0 ATP synthase subunit h	2.04
* YPL036W*	Pma2	H(+)-exporting P2-type ATPase	1.39
* YDR322C-A*	Tim11	F1F0 ATP synthase subunit e	1.35
Electron transport and membrane-associated energy conservation			
* YGR183C*	Qcr9	Ubiquinol-cytochrome-c reductase subunit 9	1.43
* YPR191W*	Qcr2	Ubiquinol-cytochrome-reductase	1.33
* YBL045C*	Cor1	Ubiquinol-cytochrome-reductase	1.34
* YGL187C*	Cox4	Cytochrome c oxidase subunit IV	1.37
* YLR038C*	Cox12	Cytochrome c oxidase subunit VIb	1.32
* YFR033C*	Qcr6	Ubiquinol-cytochrome-c reductase subunit 6	1.31
* YLR395C*	Cox8	Cytochrome c oxidase subunit VIII	1.38
Glycolysis/gluconeogenesis			
* YGR254W*	Eno1	Phosphopyruvate hydratase ENO1	1.52
* YJL052W*	Tdh1	Glyceraldehyde-3-phosphate dehydrogenase	1.84
* YGL253W*	Hxk2	Hexokinase 2	1.53
* YDL021W*	Gpm2	Phosphoglycerate mutase family	1.41
* YJR009C*	Tdh2	Glyceraldehyde-3-phosphate dehydrogenase	1.69
* YGL062W*	Pyc1	Pyruvate carboxylase 1	1.31
Pentose-phosphate shunt, oxidative branch			
* YHR183W*	Gnd1	Phosphogluconate dehydrogenase	1.64
* YHR163W*	Sol3	6-Phosphogluconolactonase	1.51

BY4741 cultivated with 5 mM vanillin for 15 h or without vanillin for 6 h when OD_600_ reached to 1.6

^a^DEPs ratio (BY4741with/without vanillin stress) which is less than 1 means down-regulation; *P*-value ≤ 0.05

Two enzymes (Gnd1 and Sol3) of the PPP increased more than 1.5-fold, particularly the enzyme Gnd1, which generates nicotinamide adenine dinucleotide phosphate (NADPH) during its catalysis [28]. PPP is the major source of NADPH, a vital cofactor for vanillin conversion to vanillyl alcohol [29–31]. This finding agrees with the finding confirmed in our previous work showing a higher NADPH/NADP^+^ cell ratio can enhance vanillin resistance [32].

**Fig. 3:**
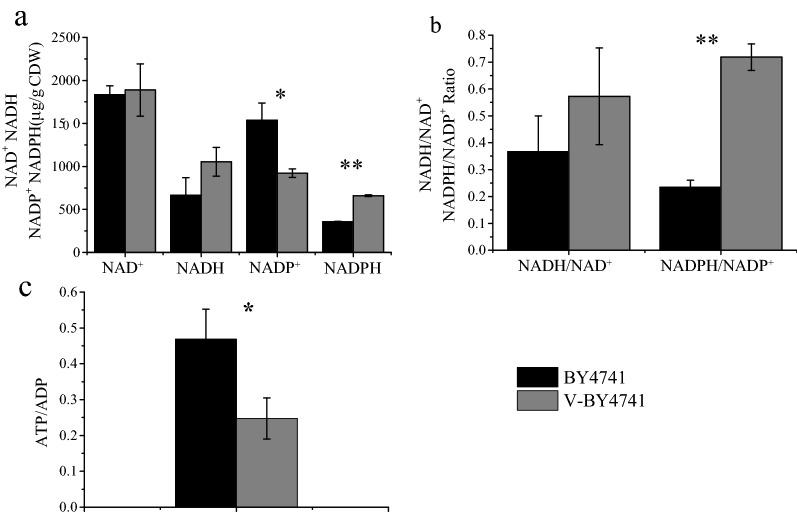
The effect of vanillin on intracellular coenzymes and ATP. NAD^+^, NADH, NADP^+^ and NADPH content. Cells were cultivated at 30 °C, 200 rpm in SC-URA liquid medium with 5 mM vanillin for 15 h or without vanillin for 6 h when OD_600_ reached to 1.6 (**a**), the ratio of NADH to NAD^+^, NADPH to NADP^+^ (**b**), the ratio of ATP to ADP (**c**). CDW, cell dry weight. V-BY4741 represents BY4741 under vanillin stress. Three independent replicates were performed for each sample. Data are the mean ± standard deviation of independent triplicate experiments. *P value < 0.05 and **P value < 0.01 in significance analysis using two-tailed Student *t* test

Our findings related to the involvement of the energy metabolism in the cellular response to vanillin. The levels of NAD(P)^+^, NAD(P)H and ATP/ADP were measured in this study (Fig. 3). The intracellular content of NADH and NADPH increased by at least 1.5- and 1.8-fold, respectively, while NAD^+^ was not obviously changed, and NADP^+^ decreased by 1.7-fold, leading to a moderately increased NADH/NAD^+^ ratio and a significantly increased NADPH/NADP^+^ ratio in vanillin-treated BY4741 cells compared with the control. These results comport with the upregulation of certain enzymes in carbon metabolism that produce more NADH and NADPH in response to vanillin stress. However, the level of ATP/ADP ratio showed a significant decrease in the presence of vanillin. This outcome was probably because the urgent requirement of vanillin detoxication causes a severe shortage of ATP, leading to the upregulation of certain enzymes involved in the glycolysis to produce as much ATP as possible to defend yeast cells from the deleterious effects of vanillin. Thus, the lower number of ribosomal proteins and ribosome biogenesis proteins may reflect the need to conserve energy, and the high activation of the glycolysis and oxidative phosphorylation pathways to produce more energy is thought to be a defense mechanism of yeast for survival in response to vanillin stress.

### **Comparative proteomics analysis after the deletion of*YRR1***

Our previous study found that vanillin repressed the ribosome biogenesis pathway. In addition, the deletion of *YRR1* can attenuate this repression and lead to enhanced resistance of *S. cerevisiae* to vanillin [12]. Thus, *YRR1* is thought to be related to ribosome biogenesis or translation pathways. However, the known *YRR1* targets are mostly related to ABC transporters and permeases, not the two aforementioned pathways. Further, the deletion of *YRR1* triggered the differential expression of only eight genes (*YBR230W-A*, *CAR2*, *FMP45*, *YCL048W-A*, *SCS3*, *UTH1*, *PMP3*, and *YIL002W-A*) at the mRNA level, and none has an effect on vanillin resistance, as shown in our previous work [12]. To investigate the protein quantity changes caused by deleting *YRR1*, a proteomic analysis was conducted with BY4741(*yrr1Δ*) and BY4741. As mentioned before, we also used 1.3-fold change as the threshold for analysis. The outcome revealed that there were 121 DEPs, including 112 upregulated and 9 downregulated proteins that showed no overlap with the levels of their corresponding mRNA. The upregulated DEPs were classified into functional categories according to the MIPS Functional Catalog and mainly fell into transcription, translation, and energy generation categories (Table 3). The functions of these differentially expressed proteins are listed in Table 4.

**Table 3:** Functional classification of upregulated proteins of *YRR1* deletion

MIPS functional category	Number of genes in category	*P*-value
RNA transport	9	1 × 10^− 5^
Transcriptional control	19	5 × 10^− 5^
Energy generation	4	3 × 10^− 4^
Electron transport	7	4 × 10^− 4^
rRNA processing	9	1 × 10^− 3^
RNA binding	9	3 × 10^− 3^
Translation elongation	3	4 × 10^− 3^
Translation termination	2	0.01

Significance was estimated with FunSpec (http://funspec.med.utoronto.ca/) based on the hypergeometric distribution of the MIPS functional categories of the differentially expressed proteins compared to the yeast proteome (P-value smaller than 0.01, bonferroni correction applied)

BY4741 and BY4741(*yrr1Δ*) cultivated in SD medium without vanillin for 6 h when OD_600_ reached to 1.6

**Table 4:** Differentially expressed proteins perturbed by the deletion of *YRR1* without vanillin stress

ORF	Protein		Description	DEPs ratio^a^
Regulation of translation				
* YOR276W*	Caf20		Phosphoprotein of the mRNA cap-binding complex	1.64
* YKL204W*	Eap1		eIF4E-associated protein	1.39
* YNL255C*	Gis2		mRNA-binding translational activator	1.40
* YCL037C*	Sro9		RNA-binding protein	1.31
* YHR087W*	Rtc3		Protein of unknown function involved in RNA metabolism	1.43
* YDR432W*	Npl3		mRNA-binding protein promotes elongation, regulates termination	1.37
* YDL053C*	Pbp4		Pbp1p binding protein	1.39
* YOL123W*	HRP1		Required for the cleavage and polyadenylation of pre-mRNA 3′ ends	1.31
* YLR150W*	Stm1		Protein required for optimal translation under nutrient stress	1.42
rRNA processing				
* YER146W*	Lsm5		Possibly involved in processing tRNA, snoRNA, and rRNA	1.31
* YDL208W*	Nhp2		snoRNA-binding protein NHP2 rRNA processing	1.42
* YGL029W*	Cgr1		Protein involved in processing of pre-rRNA	1.60
* YGR159C*	Nsr1		Pre-rRNA processing and ribosome biogenesis	1.40
* YLR192C*	Hcr1		Translation initiation factor eIF3 core subunit	1.43
* YNL124W*	Naf1		RNA-binding snoRNA assembly	1.67
* YLR068W*	Fyv7		Nucleolar protein required for maturation of 18 S rRNA	1.36
Transcription by RNA polymerase I				
* YDR174W*	Hmo1		Chromatin associated high mobility group	1.38
* YMR263W*	Sap30		Component of Rpd3L histone deacetylase complex	1.39
* YER088C*	Dot6		Protein involved in rRNA and ribosome biogenesis	1.37
* YJL148W*	Rpa34		RNA polymerase I subunit A34.5	1.47
* YDL213C*	Nop6		rRNA-binding protein required for 40 S ribosomal subunit biogenesis	1.44
* YMR260C*	Tif11		Translation initiation factor eIF1A	1.36
Nucleobase-containing compound transport				
* YOR098C*	Nup1		FG-nucleoporin NUP1	1.35
* YLR335W*	Nup2		FG-nucleoporin NUP2	1.43
* YGR119C*	Nup57		FG-nucleoporin NUP57	1.33
* YJL041W*	NSP1		FG-nucleoporin NSP1	1.55
microRNA biogenesis				
* YDL189W*	Rbs1		Assembly of the RNA polymerase III complex	1.40
Transcription factor and cofactor				
* YDR167W*		Taf10	Subunit of TFIID and SAGA complexes	1.53
* YLR399C*		Bdf1	Protein involved in transcription initiation associates with the basal transcription factor TFIID	1.46
* YKL112W*		Abf1	DNA-binding protein ABF1	1.36
* YPR133C*		Spn1	Protein involved in RNA polymerase II transcription	1.35
* YJR060W*		Cbf1	Basic helix-loop-helix (bHLH) protein	1.57
* YOR298C-A*		Mbf1	Transcriptional coactivator transcription by RNA polymerase II	1.47
* YER159C*		Bur6	Negative cofactor 2 transcription regulator complex subunit BUR6	1.38
* YBR089C-A*		Nhp6B	High-mobility group nucleosome-binding protein	1.47
* YGL025C*		Pgd1	Subunit of the RNA polymerase II mediator complex essential for basal and activated transcription	1.38
Transcription process regulation				
* YML094W*		Gim5	Facilitates transcriptional elongation	1.43
* YLR200W*		Yke2	Facilitates transcriptional elongation	1.53
Stress response				
* YLR150W*		Stm1	Protein required for optimal translation under nutrient stress	1.42
* YML007W*		Yap1	DNA-binding transcription factor YAP1	1.60
* YPR008W*		Haa1	Transcriptional activator involved in adaptation to weak acid stress	1.57
* YNL027W*		Crz1	Transcription factor, activates transcription of stress response genes	1.75
* YMR074C*		Sod2	Superoxide dismutase	1.32
* YJR104C*		Sod1	Superoxide dismutase	1.39
* YDL110C*		Tma17	ATPase dedicated chaperone that adapts proteasome assembly to stress	1.59
* YBL051C*		Pin4	Protein involved in G2/M phase progression and response to DNA damage	1.49
* YKL054C*		Def1	DNA damage-responsive RNA polymerase-degradation factor DEF1	1.65
* YFL014W*		Hsp12	Lipid-binding protein *HSP12*, induced by heat shock, oxidative stress	1.64

^a^DEPs ratio (BY4741(*yrr1Δ*)/BY4741) which is more than 1 means upregulation; *P*-value ≤ 0.05. BY4741 and BY4741(*yrr1Δ*) cultivated without vanillin for 6 h when OD_600_ reached to 1.6

### **Deletion of*YRR1*stimulated changes in the quantity changes of translational proteins**

According to the proteomic data, a series of rRNA processing proteins were upregulated after deleting *YRR1*. Among these proteins, Nop6, Nhp2, Naf1, Lsm5, Nsr1, and Fyv7 participate in the synthesis of the 40S (small) ribosomal subunit. Cgr1 is involved in processing rRNA for the 60S ribosomal subunit [33]. The factors involved in rRNA synthesis also are increased, including the subunit of RNA polymerase I Rpa34, the RNA polymerase III assembly protein Rbs1, and two transcription factors (Hmo1 and Abf1) involved in activating rRNA and ribosomal protein transcription under the control of the target of rapamycin (TOR) pathway [34–36]. Moreover, several regulators of translation, including three translation initiation factors (Tif11, Hcr1, and Gis2), were also upregulated. Considering its ability to repress ribosome genesis and translation by vanillin stress, the deletion of *YRR1* might strengthen translational elements to promote vanillin resistance in yeast.

Interestingly, the expression of three translational suppressors also increased, including that of two 4E-binding proteins, Caf20 and Eap1, which inhibit translational initiation by competing with translation initiation factor eIF4G to bind with eIF4E [37]. The other translational repressor with upregulated expression was Stm1, which was previously demonstrated to repress translational elongation by limiting the interaction of elongation factor eEF3 with ribosomes and stalling ribosomes [38]. Further, one recent study indicated that Stm1 also functions as a ribosome preservation factor in response to nutrient stress and facilitates ribosomal protein synthesis rates when nutrients levels are restored [39]. These three proteins facilitate the formation of P-bodies [40, 41]. P-bodies are viewed as reservoirs of mRNA after translation inhibition, and they are later released to reenter translation [42, 43]. It is likely that the absence of *YRR1* protects yeast cells against vanillin stress by promoting the formation of P-bodies to store translation elements and saving energy for the de novo synthesis of translation machinery.

### **The deletion of*YRR1*promotes RNA polymerase II-directed transcription**

Arbitrary extracellular and intracellular stimulation can trigger a global adaptive transcriptional regulatory program in yeast cells. The deletion of *YRR1* upregulated the expression of several basal transcription factors (Taf10, Mbf1, Nhp6b, and Pgd1). These factors facilitate recognition of promoters that contain a TATA box and assembly of the transcription preinitiation complex, thereby promoting transcription of TATA-containing genes [44–47]. TATA-containing genes tend to be responsive to stress [48, 49]. Moreover, the levels of four transcription elongation factors (Gim5, Yke2, Spn1, and Npl3) were also increased under conditions of protein abundance. Npl3, a mRNA-binding protein, is a positive transcription elongation factor shown to interact with the carboxy terminal domain (CTD) of RNA polymerase, exerting a direct stimulatory effect on the elongation activity of the polymerase [50]. These results indicate that the deletion of *YRR1* promotes TATA-containing gene transcription through the promotion of basal transcription factors to improve survival under vanillin stress.

### ***YRR1*deletion increased the protein content of Tma17, Haa1 and Mbf1, whose overexpression enhanced*S. cerevisiae*resistance to vanillin**

To search for possible novel genes/proteins for enhancing vanillin resistance, the DEPs were investigated for their functions in vanillin resistance. First, two proteins attracted our attention since they were both downregulated by vanillin stress and were upregulated upon *YRR1* deletion: Rtc3, which is involved in RNA metabolism, and plasma membrane protein Hsp12, which plays a role in maintaining membrane organization. These two proteins may be the critical for promoting the resistance of yeast to vanillin stress upon *YRR1* deletion. However, the overexpression of these proteins did not improve vanillin resistance (Additional file 1: Fig. S3).

**Fig. 4:**
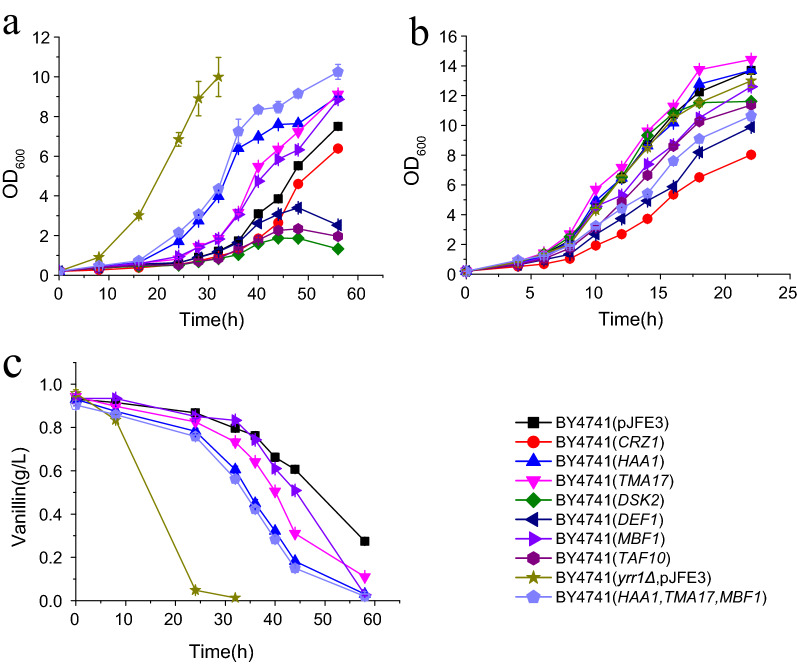
Vanillin resistance profiles of recombinant strains in SC-URA liquid medium supplemented with 6 mM vanillin (**a**) and without vanillin (**b**); the curve of vanillin consumption (**c**). Cultivations were performed at 30 °C. Samples were taken out for evaluation at indicated time points. BY4741(pJFE3) is the negative control and BY4741(*yrr1Δ*, pJFE3) is the positive control. The error bars indicated the standard deviation of independent triplicates

**Fig. 5:**
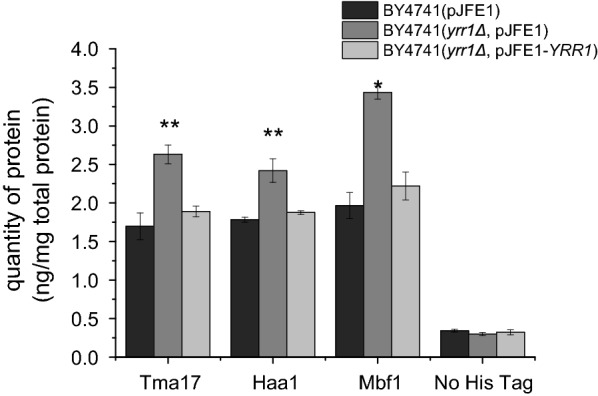
Protein levels of Tma17, Haa1 and Mbf1 in BY4741(*yrr1Δ*, pJFE1), BY4741(*yrr1Δ*, pJFE1-*YRR1*) and the control strain BY4741(pJFE1). Each protein had a 6×his tag at its C-terminal. Yeast cells were cultivated at 30 °C, 200 rpm in SC-URA liquid medium for 6 h when OD_600_ reached to 1.6. Then the cultures were collected for ELISA. Data are presented as the means ± standard errors of independent triplicate experiments. The set “No his tag” was a negative control of samples without 6×His tag for comparison. *P value < 0.001 and **P value < 0.05 in significance analysis using two-tailed Student *t* test

We investigated several upregulated proteins that were associated with transcription and the stress response in a *YRR1*-deleted strain as candidates. These candidates included five stress response-related proteins (Crz1, Dsk2, Haa1, Def1, and Tma17) and two transcription-related proteins (Taf10 and Mbf1). To determine the functions of these proteins/genes in a vanillin-resistant phenotype, their genes were overexpressed in BY4741 cells. With 5 mM vanillin treatment, the growth superiority of the recombinant strains was not as apparent as it was with 6 mM vanillin treatment. It was assumed that the contribution of a single gene was limited. An obvious resistant phenotype was observed only under severe vanillin stress. The maximum specific growth rates of the cells transformed with *HAA1*, *TMA17*, and *MBF1* were 18%, 33%, and 15% faster than the rate of the control, strain BY4741(pJFE3), in the presence of 6 mM vanillin, respectively (Fig. 4; Table 5). However, their maximum specific growth rates were more than 40% slower than those of BY4741(*yrr1Δ*, pJFE3). The specific consumption rates for vanillin in the strains overexpressing *HAA1*, *TMA17*, and *MBF1* were 0.041, 0.041, and 0.029 g g^−1^ h^−1^, respectively. Compared to the 0.034 g g^−1^ h^−1^ rate of the control strain, BY4741(pJFE3) (Table 5), only *HAA1* and *TMA17* significantly increased the specific vanillin consumption rates. *MBF1* accelerated growth to a greater extent than vanillin reduction. Compared to that contributed by *HAA1* and *TMA17*, the improvement conferred by *MBF1* was marginal. Other genes related to translation did not significantly enhance vanillin resistance (Additional file 1: Fig. S4). Then *HAA1*, *TMA17* and *MBF1* were overexpressed simultaneously in BY4741. Under 6 mM vanillin stress, the maximum specific growth rates of BY4741(*HAA1*, *TMA17*, *MBF1*) were 0.110, which was 26% higher than that of the control strain BY4741(pJFE3) but a bit lower than that of BY4741(*TMA17*). The specific consumption rates for vanillin was 32% higher than that of BY4741(pJFE3). The overexpression of multiple genes did not exhibit superimposed effect. It was probably because the overexpression of multiple genes bring burden to the cells as the result showed that the maximum specific growth rates of BY4741(*HAA1*, *TMA17*, *MBF1*) were 26% lower than that of BY4741(pJFE3) without stress. Furthermore, an ELISA was conducted with strains BY4741(*yrr1Δ*, pJFE1), BY4741(*yrr1Δ*, pJFE1-*YRR1*) and BY4741(pJFE1) in SC-URA medium without vanillin stress to determine the protein quantities of Tma17, Haa1 and Mbf1, which were replaced in situ by the corresponding genes tagged with 6×His at the C-terminal. The results showed that the protein abundance of Mbf1 (1.47-fold change in the proteome), Tma17 (1.59-fold change in the proteome) and Haa1 (1.56-fold change in the proteome) increased 1.75-, 1.55- and 1.36-fold in the strain BY4741(*yrr1Δ*, pJFE1) compared to the control strain, BY4741(pJFE1). *YRR1* was inserted into the centromere plasmid pJFE1 under the control of the *YRR1* promoter and the *PGK1* terminator to get pJFE1-*YRR1*. The pJFE1-*YRR1* was transformed into the BY4741(*yrr1Δ*) resulted in the *YRR1*-compensated strain BY4741(*yrr1Δ*, pJFE1-*YRR1*). The protein abundance of Mbf1, Tma17 and Haa1 in BY4741(*yrr1Δ*, pJFE1-*YRR1*) was decreased 35%, 28 and 22% compared to BY4741(*yrr1Δ*, pJFE1) (Fig. 5). These results indicated that the protein quantities of Haa1, Tma17 and Mbf1 are affected by *YRR1*. Besides, the growth rate of the *YRR1*-compensated strain under 6 mM vanillin stress was decreased obviously compared to according to our previous work [12]. Thus, the deletion of *YRR1* could upregulates *HAA1* and *TMA17* at the protein level to enhance vanillin resistance.

**Table 5:** Maximum specific growth rate and specific consumption rate for vanillin of recombinant strains

Strains	SC-URA medium	SC-URA with 6 mM vanillin	
	µ_max_ (h^−1^)	µ_max_ (h^−1^)	specific consumption rate for vanillin (g g^−1^ h^−1^)
BY4741(pJFE3)	0.273 ± 0.008	0.087 ± 0.005	0.034 ± 0.003
BY4741(*HAA1*)	0.252 ± 0.007	0.109 ± 0.002**	0.041 ± 0.003***
BY4741(*TMA17*)	0.280 ± 0.008	0.117 ± 0.002*	0.041 ± 0.004
BY4741(*MBF1*)	0.272 ± 0.009	0.097 ± 0.005	0.029 ± 0.004
BY4741(*HAA1, TMA17, MBF1*)	0.203 ± 0.006	0.110 ± 0.005**	0.045 ± 0.002**
BY4741(*yrr1Δ*, pJFE3)	0.267 ± 0.005	0.169 ± 0.008*	0.096 ± 0.011*

*P-value < 0.001; **P-value < 0.01; ***P-value < 0.05

The proteome analysis and comparison of CEN.PK2-1 C(*yrr1Δ*) and CEN.PK2-1 C showed that only Mbf1 exhibited an increase in protein quantity (1.32-fold change). There were 83 genes and approximately 22,000 point mutations identified as absent in CEN.PK family relative to S288C, which is derived from strain BY4741 [51, 52]. This result indicated that the different genetic background strains probably influenced the gene regulatory network.

Haa1 is a critical transcription factor involved in the acid stress adaption of yeast cells by activating the expression of *TPO2* and *TPO3* encoding two drug/H^+^-antiporters that presumably mediate the active efflux of the acetic acid [53, 54]. The overexpression of *HAA1* also contributes to vanillin resistance. But the mechanism behind it is still unknown. Mbf1 is a transcriptional coactivator that mediates transcriptional activation by bridging the DNA-binding region of an activator and a TBP (TATA-binding protein) [55]. However, recently, Liang et al. have confirmed that the TBP binding function of Mbf1 was not the reason for the enhancement of vanillin resistance mediated by Mbf1p [56]. Under stress conditions, Tma17 (also named as Adc17) is the most important chaperone for proteasome biogenesis and assembly in yeast cells. Hanssum et al. [57] found that overexpression of *TMA17* rescues 26 S proteasome defects, while deletion of *TMA17* compromises proteasome integrity and cell fitness. The maintenance of adequately functional proteasomes is vital for cells and organisms to adapt to changes and survive in their environment [57]. The overexpression of *TMA17* may increase proteasome levels or activity, and the exact mechanism needs to be analyzed in the future.

Interestingly, the deletion of *YRR1* only enhanced resistance to vanillin, not to furfural, or acetic acid, and not even to another kind of phenolic aldehyde, syringaldehyde (Additional file 1: Fig. S5). This result indicates that the enhanced resistance caused by *YRR1* deletion is vanillin-specific.

## Conclusions

Here, the changes in general protein composition perturbed by vanillin stress and *YRR1* deletion were investigated via quantitative proteome analysis. The results indicated that *S. cerevisiae* adapted to vanillin stress by repressing ribosomal protein abundance and enhancing energy production. *YRR1* deletion led to the upregulation of proteins related to the stress response and transcriptional and translational regulation, which provide unique perspectives from transcriptome analysis for comprehending the mechanisms behind the protective response of *YRR1* deletion in yeast. The finding that two proteins (Haa1 and Tma17) can enhance vanillin resistance upon their overexpression suggests novel strategies for designing inhibitor-resistant ethanologenic yeast strains for bioethanol production.

## Methods

### Strains, plasmids, and medium

*Saccharomyces cerevisiae* strain CEN.PK2-1 C *(MATa*; *ura3-52*; *trp1-289*; *leu2-3*,*112*; *his311*; *MAL2-8 C*; and *SUC2*) and BY4741 (*MATa*, *his3Δ1*, *leu2Δ0*, *met15Δ0* and *ura3Δ0*) were stored in our laboratory. The *YRR1*-deleted strain CEN.PK2-1 C(*yrr1Δ*) (CEN.PK2-1 C derivative; *yrr1::loxP*) and BY4741(*yrr1Δ*) (BY4741 derivative; *yrr1::loxP*) [12] that were previously stored and constructed in our laboratory, respectively. The laboratory strain BY4741 was engineered for gene overexpression in this study. The genes *CRZ1*, *HAA1*, *TMA17*, *DSK2*, *DEF1*, *MBF1*, *TAF10*, *RTC3*, *HSP12*, *CAF20* and *TIF11* were amplified by polymerase chain reaction (PCR) using the genomic DNA of *S. cerevisiae* BY4741 as the template. The PCR products were gel-purified, and *DEF1*, *DSK2* and *CRZ1* were digested by BamHI and SalI, and the other genes were digested by BamHI and PstI. Then, the genes were ligated into plasmid pJFE3 predigested with BamHI and SalI or PstI. Plasmid pJFE3 is a 2-*μ* plasmid with the *TEF1* promoter, the *PGK1 *terminator, and *URA3* as a selection marker [58]. To overexpress of multiple genes (*HAA1*, *TMA17* and *MBF1*) in one strain, the *MBF1* was cloned into the 2-*μ* plasmid pIYC04 [59] under the control of the *TEF1* promoter and the *ADH1* terminator with NotI and SacI. The *TMA17* was cloned into plasmid pIYC04 under the control of the *PGK1* promoter and the *CYC1* terminator with BamHI and SacI. Then the expression cassettes of *TMA17* and *MBF1* were cloned in to the plasmid pJFE3-*HAA1* (*HAA1* was cloned into pJFE3) using Gibson assembly. Next, the resulting recombinant plasmids were transformed into BY4741. The open reading frames of *HAA1*, *MBF1*, *TMA17* were tagged at their natural chromosomal locations with six-His epitopes encoding proteins with C-terminally tags. Tagging was performed using homologous recombination as described in our previous report [12]. The expression cassette containing *LoxP–KanMX4–LoxP* cassettes and 6×His-tagged homologous sequence with target-genes were transformed into the strains BY4741 and BY4741(*yrr1Δ*), and the mutants were screened in YPD or SC-URA medium with 800 mg L^−1^ G418. All strains and primers employed in this study are listed in Additional file 1: Tables S1 and S2, respectively. The *YRR1* was cloned into the centromere plasmid pJFE1 and under the control of the *YRR1* promoter and the *PGK1* terminator. The *TEF1* promoter of pJFE1 was replaced by the *YRR1* promoter, producing the plasmid pJFE1-*YRR1*. Then the plasmid pJFE1-*YRR1* was transformed into BY4741(*yrr1Δ*) to get the *YRR1*-compensated strain BY4741(*yrr1Δ*, pJFE1-*YRR1*).

Synthetic complete medium (SD) or SC-URA medium (5 g L^−1^ ammonium sulfate, Sangon, China, 1.7 g L^−1^ yeast nitrogen base without amino acids, Sangon, China, CSM or CSM-URA, MP Biomedicals, Solon, OH, USA) was used for activation, batch fermentation, and protein sample preparation of recombinant strains upon the addition of 20 g L^−1^ glucose. Vanillin was added to the medium as indicated. All of the cultures were incubated at 30 °C.

### Protein sample preparation for proteomic analysis

Yeast cells of strains BY4741, BY4741(*yrr1Δ*), CEN.PK2-1C and CEN.PK2-1C(*yrr1Δ*) were harvested when the OD_600_ reached approximately 1.6 in logarithmic phase after different times in culture with SD medium with or without of 5 mM vanillin. All of the proteomic analyses were carried out as biological triplicates. The sample was ground by liquid nitrogen into cell powder and then transferred to a 5-mL centrifuge tube. Four volumes of lysis buffer (8 M urea, 1% Triton X-100, 10 mM dithiothreitol, 2 mM EDTA and 1% protease inhibitor cocktail) were added to the cell powder, sonicated three times on ice using a high-intensity ultrasonic processor. The debris was removed by centrifugation at 12,000×*g* for 10 min at 4 °C. Finally, the supernatant was collected, and the protein concentration was determined using a BCA kit (Beyotime, P0010S, Shanghai, China) according to the manufacturer’s instructions. For digestion, the protein solution was reduced with 5 mM dithiothreitol for 30 min at 56 °C and alkylated with 11 mM iodoacetamide for 15 min at room temperature in darkness. The protein sample was then diluted by adding 100 mM triethylammonium bicarbonate (TEAB) to reduce the urea concentration to < 2 M. Finally, trypsin was added at a 1:50 trypsin-to-protein mass ratio for the first digestion overnight; the second 4-h digestion process was based on a 1:100 trypsin-to-protein mass ratio. A trypsin digestion peptide was labeled with a tandem mass tag (TMT), according to the TMT kit manufacturer’s protocol (Thermo Fisher Scientific, 90068, Waltham, USA).

### High-performance liquid chromatography fractionation and mass spectrometric analysis

The labeled peptides were fractionated by high pH reverse-phase high-performance liquid chromatography (HPLC) using an Agilent 300 Extend C18 column (5-µm particles, 4.6-mm ID, and 250-mm length). Briefly, the peptides were first separated into 60 fractions with a gradient ranging from 8 to 32% acetonitrile (pH 9.0) for more than 60 min. Then, the peptides were combined into 18 fractions and dried by vacuum centrifugation. The tryptic peptides were dissolved in solvent A (0.1% formic acid and 2% acetonitrile); the gradient was composed of an increase in solvent B (0.1% formic acid in 90% acetonitrile) from 6 to 20% over 20 min, from 20 to 33% in 11 min, to 80% in 4 min and then holding at 80% for the last 3 min, all at a constant flow rate of 320 nL min^−1^ on an EASY-nLC 1000 UPLC system (Thermo Fisher Scientific, EASY-nLC 1000, Waltham, USA). The peptides were subjected to a nano-electrospray ionization (NSI) source followed by tandem mass spectrometry (MS/MS) in a Q Exactive™ Plus (Thermo) coupled online to a UPLC. The applied electrospray voltage applied was 2.0 kV. The m/z scan range was 350–1800 for a full scan, and intact peptides were detected in an Orbitrap at a resolution of 70,000. The secondary mass spectrometry scan range was set as a fixed first point of 100 m/z, and the resolution was 17,500. The data acquisition mode was based on a data-dependent scanning (DDA) program; the first 20 peptides with the highest signal intensity were selected to enter the high energy collision dissociation (HCD) collision cell, and then 30% of the fragmentation energy was used for fragmentation after the first-stage scanning; the same step was performed again during the secondary mass spectrometry analysis. To improve the effective utilization of the mass spectrometer, the automatic gain control (AGC) was set as 5E4, the signal threshold was set as 1E4, and the maximum injection time was set as 200 ms with 30 s of dynamic exclusion time.

### Database searching and data analysis

The resulting MS/MS data were processed by using the MaxQuant search engine (v.1.5.2.8), a platform for mass spectrometry (MS)-based proteomic data analysis, to match the secondary mass spectrum with the theoretical spectrum in the protein database. The search parameters were as follows: trypsin/P was specified as the cleavage enzyme and to two missing cleavages were allowed; the mass resistance for the precursor ions was set as 20 ppm for the first search and 5 ppm for the main search, and the mass resistance for fragment ions was set as 0.02 Da. Carbamidomethyl on Cys was specified asa fixed modification, and oxidation of Met was specified as a variable modification. The method used to obtain relative protein expression ratios was set as TMT-6plex, since relative quantification of peptides/proteins was carried out based on the signal intensity of the reporter ions in the TMT tag fragment, and the false discovery rate (FDR) for protein identification and peptide spectrum match (PSM) identification was 1% eliminate false positive peptide matching. Proteins with an average increase or decrease ratio > 1.3-fold were classified into functional categories by GO annotation based on the Uniprot-GOA database (http://www.ebi.ac.uk/GOA/). Some undetermined proteins were identified using protein sequence-based algorithm software (InterProScan) to predict the GO function of the protein. Pathway enrichment analyses were carried out using the Kyoto Encyclopedia of Genes and Genomes (KEGG) database, and the subcellular location of the proteins was determined using software WoLF PSORT software. The function of representative proteins was estimated using the MIPS Functional Catalog database (http://funspec.med.utoronto.ca/, *P-*value < 0.01, Bonferroni correction). To explore the relationship between the proteome and the transcriptome, the relationship of them was contrasted and verified by utilizing the Statistical Package for the Social Sciences (SPSS) software (Spearman’s correlation analysis), and their overlap is displayed in a Venn diagram generated using a bioinformatic tool (http://bioinformatics.psb.ugent.be/webtools/Venn/).

### Fermentation

A single colony was precultured in 3 mL of SC-URA at 30 °C for 24 h. Then, each culture was transferred into 10 mL of fresh medium with an initial OD_600_ of 0.2 and cultured overnight. Next, the cells were inoculated into 100-mL flasks containing 40 mL of fermentation medium in the presence or absence of 6 mM vanillin with an initial OD_600_ of 0.2. The fermentation was carried out at 30 °C and 200 rpm.

### Analysis of extracellular vanillin

The concentrations of vanillin and vanillyl alcohol were determined by a HPLC Waters system e2695 (Waters, USA) equipped with an Xbridge^TM^-C18 column (Waters, USA). The peaks were detected at room temperature using ultraviolet detector (PDA-2998) at 210 nm with a mobile phase containing 40% absolute methanol (Chromatographic grade, Fisher Chemical, USA) supplied at a flow rate of 0.6 mL min^−1^ [12].

### Growth and vanillin consumption assays

The maximum growth rates were regarded as the linear regression coefficients of the ln (OD_600_) versus time during the exponential growth phase [60]. The specific consumption rates of vanillin were calculated using the following equation:

$$r = \frac{{An - Am}}{{\frac{1}{2}\sum\limits_{i = m + 1}^n {\left( {{B_i} + {B_{i - 1}}} \right) \times \left( {{t_i} - {t_{i - 1}}} \right)} }},$$

where *r* is the specific consumption rate during the phase from sampling point *m* to sampling point *n*; *A*, *B*, and *t* are the metabolite concentration, biomass concentration, and time, respectively, at sampling points *n*, *i*, and *m*, as previously described [12, 61]. The biomass concentrates were investigated to determine their correlation with OD_600_ and the dry cell weight (DCW). Different strains exhibited different coefficients between OD_600_ and DCW.

### NAD(P)^+^, NAD(P)H and ATP/ADP assays

Cells were cultured in 40 mL of SC-URA medium with or without 6 mM vanillin in a 100-mL flask starting at an initial OD_600_ of 0.2. Rapid sampling, quenching, and metabolite extraction of biomass (in approximately 10 mL of culture at exponential phase) for the NAD(P)^+^ and NAD(P)H assays were performed as described previously [61]. Subsequently, the concentration of the resultant extract was quantified using a sensitive enzymatic cycling assay as previously reported [28, 62].

Samples of 1 mL of culture were taken for intracellular ATP/ADP ratio analysis and quenched by liquid nitrogen. The yeast cells were suspended in 0.5 mL of 0.6% (w/w) HClO_4_, after which cell extraction was vigorously mixied in a freezing grinder (Jin Xing, China) for 10 min. The cellular debris was removed by centrifugation at 13,000 rpm for 10 min and subsequent extracts were neutralized with 3 M KOH (pH 7.0). The ratio of ATP/ADP in the extracts was determined using an ADP/ATP ratio assay kit (Sigma, USA) according to the manufacturer’s instructions. In summary, first, ATP immediately reacted with the luciferase substrate d-luciferin to produce light, and the luminescence was measured (relative light units, RLU) on a luminometer for an ATP assay (RLUA). Then, after 10 min of incubation, luminescence was measured for ATP (RLUB) in a background. In the second step, an ADP reagent was added to each well, ADP was converted to ATP through an enzymatic reaction and then the resulting product was allowed to react with the d-luciferin as in the first step, which was measured as RLUC. The ATP/ADP ratio was calculated using the following equation:

$${{{\text{ATP}}} \mathord{\left/ {\vphantom {{{\text{ATP}}} {{\text{AD}}{{\text{P}}_{{\text{ratio}}}}}}} \right. \kern-\nulldelimiterspace} {{\text{AD}}{{\text{P}}_{{\text{ratio}}}}}} = \frac{{{\text{RLUA}}}}{{{\text{RLUC}} - {\text{RLUB}}}}$$

Three biological replicate experiments were performed, and the results are expressed as the mean and standard deviation (SD). Student’s *t* test was used for statistical analyses with significance levels of *P < 0.05, **P < 0.01.

### His-tag ELISA detection

Haa1, Mbf1 or Tma17 was tagged with 6×His at the C-terminus using in situ replacement technology. The tagged cells were cultured in a 500-mL flask with 200 mL of SD medium to an OD_600_ of 1.5-2. Protein extraction was performed according to a previous study with minor modifications [63]. In brief, harvested yeast cells were pelleted and washed with ice-cold distilled water. Cells were resuspended in an equal volume of cold disruption buffer (20 mM Tris, pH 7.9, 10 mM MgCl_2_, 1 mM EDTA, 5% glycerol, 1 mM DTT, 0.3 M ammonium sulfate and 1 mM PMSF) and added 0.6–0.8 g of chilled glass beads were added. Subsequently, the debris in the lysate was removed by centrifugation. The total amount of protein in the protein extracts was determined using BCA protein assay reagent kit (Beyotime, China). The amounts of Haa1, Mbf1 and Tma17 in the total protein extracts were qualified using a the His-tag ELISA detection kit (GenScript, China) based on competitive ELISA, as described in the manufacturer’s instructions described. The His-tag standards or samples in this study containing His-tagged proteins in this study were incubated on a microwell plate for competitive with an anti-His tag mAb and subsequently allowed to react with horseradish peroxidase (HRP)-conjugated antibody. The tetramethylbenzidine (TMB) substrate was catalyzed by HRP and produced a blue product that was measured in a microplate reader (Bio-Tek, USA). Standard curves were constructed using a gradient concentration of His-tag standards. The levels of Haa1, Mbf1 and Tma17 in the strains were calculated as (the amount of Haa1, Mbf1 and Tma17)/(the total amount of protein).

## Supplementary Information

**Additional file 1: Table S1.** Yeast strains used in this study. **Table S2.** List of primers used for plasmids and strain construction in this work. **Figure S1.** Influence of vanillin stress on KEGG pathway enrichment of down-regulated DEPs in CEN.PK2-1C background strains. Greater RichFactor indicates a greater effect of vanillin on the analyzed pathway. **Figure S2.** Influence of vanillin stress on KEGG pathway enrichment of upregulated DEPs in CEN.PK2-1C background strains. Greater RichFactor indicates a greater effect of vanillin on the analyzed pathway. **Figure S3.** Growth curve of overexpression of *RTC3*, *HSP12* in presence of 6 mmol L^−1^ vanillin. BY4741(pJFE3) and BY4741(*yrr1Δ*, pJFE3) are the controls. Data are presented as the means ± standard errors of independent triplicate experiments. **Figure S4.** Growth curve of strains overexpressing translation related proteins encoding genes *CAF20*, *LSM5*, *CGR1*, *GIS2*, *TIF11* cultured in SC-URA with 6 mmol L^−1^ vanillin. BY4741(pJFE3) and BY4741(*yrr1Δ*, pJFE3) are the controls. Data are presented as the means ± standard errors of independent triplicate experiments. **Figure S5.** Spot growth of BY4741(*yrr1Δ*) with different inhibitors.

## Data Availability

The proteomic data generated in this study has been deposited to the ProteomeXchange consortium (http://proteomecentral.proteomexchange.org) with the dataset identifier PXD017738 and PXD020951. Other datasets used and/ or analyzed in this study are available from the corresponding author upon reasonable request.

## Abbreviations

mAb: Monoclonal antibody
HRP: Horseradish peroxidase
HMF: Hydroxymethyl furfural
P-bodies: Processing bodies
SGs: Stress granules
HPLC: High-performance liquid chromatography
DDA: Data-dependent scanning
AGC: Automatic gain control
KEGG: Kyoto Encyclopedia of Genes and Genomes
SPSS: Social Sciences software
DCW: Dry cell weight
GO: Gene Ontology
DEPs: Differentially expressed proteins
DEGs: Differentially expressed genes
Sr: Spearman rank
PPP: Pentose phosphate pathway
ATP: Adenosine triphosphatase
ABC: ATP binding cassette
NADPH: Nicotinamide adenine dinucleotide phosphate
ADH: Alcohol dehydrogenase
TMB: Tetramethylbenzidine
TEAB: Triethylammonium bicarbonate
TMT: Tandem Mass Tag
HPLC: High-performance liquid chromatography
NSI: Nano electrospray ionization source
HCD: High energy collision dissociation
AGC: Automatic gain control
FDR: False discovery rate
PSM: Peptide spectrum match

## References

[1] Gibson BR, Lawrence SJ, Leclaire JPR, Powell CD, Smart KA (2007). Yeast responses to stresses associated with industrial brewery handling. FEMS Microbiol Rev.

[2] Hong K-K, Nielsen J (2012). Metabolic engineering of *Saccharomyces cerevisiae*: a key cell factory platform for future biorefineries. Cell Mol Life Sci.

[3] Kim S, Dale BE (2004). Global potential bioethanol production from wasted crops and crop residues. Biomass Bioenergy.

[4] Saini JK, Saini R, Tewari L (2015). Lignocellulosic agriculture wastes as biomass feedstocks for second-generation bioethanol production: concepts and recent developments. 3 Biotech.

[5] Klinke HB, Thomsen AB, Ahring BK (2004). Inhibition of ethanol-producing yeast and bacteria by degradation products produced during pre-treatment of biomass. Appl Microbiol Biotechnol.

[6] Almeida JRM, Modig T, Petersson A, Hähn-Hägerdal B, Lidén G, Gorwa-Grauslund MF (2007). Increased tolerance and conversion of inhibitors in lignocellulosic hydrolysates by *Saccharomyces cerevisiae*. J Chem Technol Biotechnol.

[7] Heer D, Sauer U (2008). Identification of furfural as a key toxin in lignocellulosic hydrolysates and evolution of a tolerant yeast strain. Microb Biotechnol.

[8] Brochado AR, Matos C, Moller BL, Hansen J, Mortensen UH, Patil KR (2010). Improved vanillin production in baker’s yeast through in silico design. Microb Cell Fact.

[9] Hansen EH, Møller BL, Kock GR, Bünner CM, Kristensen C, Jensen OR, Okkels FT, Olsen CE, Motawia MS, Hansen J (2009). De novo biosynthesis of vanillin in fission yeast (*Schizosaccharomyces pombe*) and baker’s yeast (*Saccharomyces cerevisiae*). Appl Environ Microb.

[10] Li X, Zheng Y (2020). Biotransformation of lignin: mechanisms, applications and future work. Biotechnol Prog.

[11] Liang C, Zhang X, Wu J, Mu S, Wu Z, Jin JM, Tang SY (2020). Dynamic control of toxic natural product biosynthesis by an artificial regulatory circuit. Metab Eng.

[12] Wang X, Liang Z, Hou J, Shen Y, Bao X (2017). The absence of the transcription factor Yrr1p, identified from comparative genome profiling, increased vanillin tolerance due to enhancements of ABC transporters expressing, rRNA processing and ribosome biogenesis in *Saccharomyces cerevisiae*. Front Microbiol.

[13] Iwaki A, Ohnuki S, Suga Y, Izawa S, Ohya Y (2013). Vanillin inhibits translation and induces messenger ribonucleoprotein (mRNP) granule formation in *Saccharomyces cerevisiae*: application and validation of high-content, image-based profiling. PLoS ONE.

[14] Iwaki A, Kawai T, Yamamoto Y, Izawa S (2013). Biomass conversion inhibitors furfural and 5-hydroxymethylfurfural induce formation of messenger RNP granules and attenuate translation activity in *Saccharomyces cerevisiae*. Appl Environ Microb.

[15] Iwaki A, Izawa S (2012). Acidic stress induces the formation of P-bodies, but not stress granules, with mild attenuation of bulk translation in *Saccharomyces cerevisiae*. Biochem J.

[16] Wu B, Qiao J, Wang X, Liu M, Xu S, Sun D (2021). Factors affecting the rapid changes of protein under short-term heat stress. BMC Genom.

[17] De Groot MJ, Daran-Lapujade P, van Breukelen B, Knijnenburg TA, de Hulster EA, Reinders MJ, Pronk JT, Heck AJ, Slijper M (2007). Quantitative proteomics and transcriptomics of anaerobic and aerobic yeast cultures reveals post-transcriptional regulation of key cellular processes. Microbiology.

[18] Li P, Fu X, Chen M, Zhang L, Li S (2019). Proteomic profiling and integrated analysis with transcriptomic data bring new insights in the stress responses of Kluyveromyces marxianus after an arrest during high-temperature ethanol fermentation. Biotechnol Biofuels.

[19] Deepak K, Varshney S, Sengupta S, Sharma N (2019). A comparative study of the proteome regulated by the Rpb4 and Rpb7 subunits of RNA polymerase II in fission yeast. J Proteom.

[20] The Gene Ontology C (2019). The gene ontology resource: 20 years and still GOing strong. Nucleic Acids Res.

[21] Kanehisa M, Furumichi M, Tanabe M, Sato Y, Morishima K (2017). KEGG: new perspectives on genomes, pathways, diseases and drugs. Nucleic Acids Res.

[22] Nguyen TTM, Aya I, Shingo I (2015). The *ADH7 *promoter of *Saccharomyces cerevisiae*is vanillin-inducible and enables mRNA translation under severe vanillin stress. Front Microbiol.

[23] Gerhardy S, Menet AM, Peña C, Petkowski JJ, Panse VG (2014). Assembly and nuclear export of pre-ribosomal particles in budding yeast. Chromosoma.

[24] Warner JR (1999). The economics of ribosome biosynthesis in yeast. Trends Biochem Sci.

[25] Albert B, Kos-Braun IC, Henras AK, Dez C, Rueda MP, Zhang X, Gadal O, Kos M, Shore D (2019). A ribosome assembly stress response regulates transcription to maintain proteome homeostasis. Elife.

[26] Bianconi ML (2003). Calorimetric determination of thermodynamic parameters of reaction reveals different enthalpic compensations of the yeast hexokinase isozymes. J Biol Chem.

[27] Randez-Gil F, Sanz P, Entian K-D, Prieto JA (1998). Carbon source-dependent phosphorylation of hexokinase PII and its role in the glucose-signaling response in yeast. Mol Cell Biol.

[28] Sinha A, Maitra PK (1992). Induction of specific enzymes of the oxidative pentose phosphate pathway by glucono-δ-lactone in *Saccharomyces cerevisiae*. Microbiology.

[29] Cunha JT, Aguiar TQ, Romani A, Oliveira C, Domingues L (2015). Contribution of *PRS3*, *RPB4* and *ZWF1* to the resistance of industrial *Saccharomyces cerevisiae* CCUG53310 and PE-2 strains to lignocellulosic hydrolysate-derived inhibitors. Bioresour Technol.

[30] Liu ZL (2011). Molecular mechanisms of yeast tolerance and in situ detoxification of lignocellulose hydrolysates. Appl Microbiol Biotechnol.

[31] Shen Y, Li H, Wang X, Zhang X, Hou J, Wang L, Gao N, Bao X (2014). High vanillin tolerance of an evolved *Saccharomyces cerevisiae* strain owing to its enhanced vanillin reduction and antioxidative capacity. J Ind Microbiol Biotechnol.

[32] Wang X, Liang Z, Hou J, Bao X, Shen Y (2016). Identification and functional evaluation of the reductases and dehydrogenases from *Saccharomyces cerevisiae* involved in vanillin resistance. BMC Biotechnol.

[33] Moy TI, Boettner D, Rhodes JC, Silver PA, Askew DS (2002). Identification of a role for *Saccharomyces cerevisiae* Cgr1p in pre-rRNA processing and 60S ribosome subunit synthesis. Microbiology.

[34] Berger AB, Decourty L, Badis G, Nehrbass U, Jacquier A, Gadal O (2007). Hmo1 is required for TOR-dependent regulation of ribosomal protein gene transcription. Mol Cell Biol.

[35] Oliveira AP, Ludwig C, Zampieri M, Weisser H, Aebersold R, Sauer U (2015). Dynamic phosphoproteomics reveals TORC1-dependent regulation of yeast nucleotide and amino acid biosynthesis. Sci Signal.

[36] Fermi B, Bosio MC, Dieci G (2017). Multiple roles of the general regulatory factor Abf1 in yeast ribosome biogenesis. Curr Genet.

[37] Castelli LM, Talavera D, Kershaw CJ, Mohammad-Qureshi SS, Costello JL, Rowe W, Sims PF, Grant CM, Hubbard SJ, Ashe MP (2015). The 4E-BP Caf20p mediates both eIF4E-dependent and independent repression of translation. PLoS Genet.

[38] Balagopal V, Parker R (2011). Stm1 modulates translation after 80S formation in *Saccharomyces cerevisiae*. RNA.

[39] Hayashi H, Nagai R, Abe T, Wada M, Ito K, Takeuchi-Tomita N (2018). Tight interaction of eEF2 in the presence of Stm1 on ribosome. J Biochem.

[40] Park K, Lee Y-S, Jung D, Kim J (2018). Roles of eIF4E-binding protein Caf20 in Ste12 translation and P-body formation in yeast. J Microbiol.

[41] Balagopal V, Parker R (2009). Stm1 modulates mRNA decay and Dhh1 function in *Saccharomyces cerevisiae*. Genetics.

[42] Brengues M, Teixeira D, Parker R (2005). Movement of eukaryotic mRNAs between polysomes and cytoplasmic processing bodies. Science.

[43] Standart N, Weil D (2018). P-bodies: cytosolic droplets for coordinated mRNA storage. Trends Genet.

[44] Matangkasombut O, Buratowski RM, Swilling NW, Buratowski S (2000). Bromodomain factor 1 corresponds to a missing piece of yeast TFIID. Gene Dev.

[45] Robinson PJ, Trnka MJ, Bushnell DA, Davis RE, Mattei P-J, Burlingame AL, Kornberg RD (2016). Structure of a complete mediator-RNA polymerase II pre-initiation complex. Cell.

[46] Han Y, Luo J, Ranish J, Hahn S (2014). Architecture of the *Saccharomyces cerevisiae* SAGA transcription coactivator complex. EMBO J.

[47] Sermwittayawong D, Tan S (2006). SAGA binds TBP via its Spt8 subunit in competition with DNA: implications for TBP recruitment. EMBO J.

[48] López-Maury L, Marguerat S, Bähler J (2008). Tuning gene expression to changing environments: from rapid responses to evolutionary adaptation. Nat Rev Genet.

[49] Bae S-H, Han HW, Moon J (2015). Functional analysis of the molecular interactions of TATA box-containing genes and essential genes. PLoS ONE.

[50] Dermody JL, Dreyfuss JM, Villén J, Ogundipe B, Gygi SP, Park PJ, Ponticelli AS, Moore CL, Buratowski S, Bucheli ME (2008). Unphosphorylated SR-like protein Npl3 stimulates RNA polymerase II elongation. PLoS ONE.

[51] Nijkamp JF, van den Broek M, Datema E, de Kok S, Bosman L, Luttik MA (2012). De novo sequencing, assembly and analysis of the genome of the laboratory strain *Saccharomyces cerevisiae* CEN.PK113-7D, a model for modern industrial biotechnology. Microb Cell Fact.

[52] Espinosa MI, Williams TC, Pretorius IS, Paulsen IT (2019). Benchmarking two *Saccharomyces cerevisiae* laboratory strains for growth and transcriptional response to methanol. Synth Syst Biotechnol.

[53] Swinnen S, Henriques SF, Shrestha R, Ho P-W, Sá-Correia I, Nevoigt E (2017). Improvement of yeast tolerance to acetic acid through Haa1 transcription factor engineering: towards the underlying mechanisms. Microb Cell Fact.

[54] Fernandes AR, Mira NP, Vargas RC, Canelhas I, Sá-Correia I (2005). *Saccharomyces cerevisiae* adaptation to weak acids involves the transcription factor Haa1p and Haa1p-regulated genes. Biochem Biophys Res Commun.

[55] Liu Q-X, Nakashima-Kamimura N, Ikeo K, Hirose S, Gojobori T (2007). Compensatory change of interacting amino acids in the coevolution of transcriptional coactivator *MBF1* and TATA-Box–binding protein. Mol Biol Evol.

[56] Liang Z, Wang X, Bao X, Wei T, Hou J, Liu W (2020). Newly identified genes contribute to vanillin tolerance in *Saccharomyces cerevisiae*. Microb Biotechnol.

[57] Hanssum A, Zhong Z, Rousseau A, Krzyzosiak A, Sigurdardottir A, Bertolotti A (2014). An inducible chaperone adapts proteasome assembly to stress. Mol Cell.

[58] Shen Y, Chen X, Peng BY, Chen LY, Hou J, Bao XM (2012). An efficient xylose-fermenting recombinant *Saccharomyces cerevisiae* strain obtained through adaptive evolution and its global transcription profile. Appl Microbiol Biotechnol.

[59] Chen Y, Daviet L, Schalk M, Siewers V, Nielsen J (2013). Establishing a platform cell factory through engineering of yeast acetyl-CoA metabolism. Metab Eng.

[60] Peng B, Shen Y, Li X, Chen X, Hou J, Bao X (2012). Improvement of xylose fermentation in respiratory-deficient xylose-fermenting *Saccharomyces cerevisiae*. Metab Eng.

[61] Ask M, Bettiga M, Mapelli V, Olsson L (2013). The influence of HMF and furfural on redox-balance and energy-state of xylose-utilizing *Saccharomyces cerevisiae*. Biotechnol Biofuels.

[62] Zhou Y, Wang L, Yang F, Lin X, Zhang S, Zhao ZK (2011). Determining the extremes of the cellular NAD(H) level by using an *Escherichia coli* NAD(+)auxotrophic mutant. Appl Environ Microbiol.

[63] Akache B, Macpherson S, Sylvain MA, Turcotte B (2004). Complex interplay among regulators of drug resistance genes in *Saccharomyces cerevisiae*. J Biol Chem.

