# Supplementary material for: Proteomic analysis revealed the roles of YRR1 deletion in enhancing the vanillin resistance of Saccharomyces cerevisiae
Source: Microb Cell Fact. 2021 Jul 23;20:142. doi: 10.1186/s12934-021-01633-z (PMC8305865; doi:10.1186/s12934-021-01633-z)
Supplement: Supplementary file 1 — Additional file 1: Table S1. Yeast strains used in this study. Table S2. List of primers used for plasmids and strain construction in this work. Figure S1. Influence of vanillin stress on KEGG pathway enrichment of down-regulated DEPs in CEN.PK2-1C background strains. Greater RichFactor indicates a greater effect of vanillin on the analyzed pathway. Figure S2. Influence of vanillin stress on KEGG pathway enrichment of upregulated DEPs in CEN.PK2-1C background strains. Greater RichFactor indicates a greater effect of vanillin on the analyzed pathway. Figure S3. Growth curve of overexpression of RTC3, HSP12 in presence of 6 mmol L−1 vanillin. BY4741(pJFE3) and BY4741(yrr1Δ, pJFE3) are the controls. Data are presented as the means ± standard errors of independent triplicate experiments. Figure S4. Growth curve of strains overexpressing translation related proteins encoding genes CAF20, LSM5, CGR1, GIS2, TIF11 cultured in SC-URA with 6 mmol L−1 vanillin. BY4741(pJFE3) and BY4741(yrr1Δ, pJFE3) are the controls. Data are presented as the means ± standard errors of independent triplicate experiments. Figure S5. Spot growth of BY4741(yrr1Δ) with different inhibitors. [file 12934_2021_1633_MOESM1_ESM.docx]

# Additional file 1

**Proteomic analysis revealed the roles of *YRR1* deletion in enhancing the vanillin resistance of *Saccharomyces cerevisiae***

Wenyan Cao^1#^, Weiquan Zhao^1#^, Bolun Yang^1^, XinningWang^1^*, Yu Shen^2^, Tiandi Wei^2^, Wensheng Qin^3^, Zailu Li^1^, Xiaoming Bao^1^

* Corresponding author: Dr. Xinning Wang；email: [xnwang@qlu.edu.cn](mailto:xnwang@qlu.edu.cn)

^#^ Wenyan Cao and Weiquan Zhao shared first authors.

^1^State Key Laboratory of Biobased Material and Green Papermaking, School of Bioengineering, Key Laboratory of Shandong Microbial Engineering, Qilu University of Technology (Shandong Academy of Sciences), 3501 Daxue Road, Jinan, 250353, China.

^2^State Key Laboratory of Microbial Technology, Institute of Microbial Technology, Shandong University, Binhai Road 72, Qingdao, 266237, China.

^3^Department of Biology, Lakehead University, 955 Oliver Rd, Thunder Bay, ON, P7B 5E1, Canada.

Table S1 Yeast strains used in this study

| Strains | Properties | Source/reference |
| --- | --- | --- |
| BY4741 | *S. cerevisiae* laboratory strain, *MATα his3-∆1 leu2-∆0 met15-∆0 ura3-∆0* | Euroscarf |
| BY4741(*yrr1Δ*) | BY4741 derivate; yrr1::loxP | Lab preservation |
| CEN.PK2-1C | *MATa*; *ura3-52*; *trp1-289*; *leu2-3*,*112*; *his311*; *MAL2-8C*; *SUC2* | Euroscarf |
| CEN.PK2-1C(*yrr1Δ*) | CEN.PK2-1C derivate; yrr1::loxP | Lab preservation |
| BY4741(pJFE3) | BY4741 carrying empty plasmid pJFE3 | Lab preservation |
| BY4741(*CRZ1*) | BY4741 with *CRZ1* overexpression | This study |
| BY4741(*DSK2*) | BY4741 with *DSK2* overexpression | This study |
| BY4741(*HAA1*) | BY4741 with *HAA1* overexpression | This study |
| BY4741(*DEF1*) | BY4741 with DEF1 overexpression | This study |
| BY4741(*TMA17*) | BY4741 with *TMA17* overexpression | This study |
| BY4741(*TAF10*) | BY4741 with *TAF10* overexpression | This study |
| BY4741(*MBF1*) | BY4741 with *MBF1* overexpression | This study |
| BY4741(*CAF20*) | BY4741 with *CAF20* overexpression | This study |
| BY4741(*CGR1*) | BY4741 with *CGR1* overexpression | This study |
| BY4741(*GIS2*) | BY4741 with *GIS2* overexpression | This study |
| BY4741(*TIF11*) | BY4741 with *TIF11* overexpression | This study |
| BY4741(*LSM5*) | BY4741 with *LSM5* overexpression | This study |
| BY4741(*HAA1*-6×his, pJFE1) | The C-terminally of Haa1 was tagged with six His epitopes at their natural chromosomal locations in BY4741(pJFE1) | This study |
| BY4741(*TMA17*-6×his, pJFE1) | The C-terminally of Tma17 was tagged with six His epitopes at their natural chromosomal locations in BY4741(pJFE1) | This study |
| BY4741(*MBF1*-6×his, pJFE1) | The C-terminally of Mbf1 was tagged with six His epitopes at their natural chromosomal locations in BY4741(pJFE1) | This study |
| BY4741(*yrr1Δ, HAA1-6*×his, pJFE1) | The C-terminally of Haa1 was tagged with six His epitopes at their natural chromosomal locations in BY4741(*yrr1Δ,* pJFE1) | This study |
| BY4741(*yrr1Δ, MBF1*-6×his, pJFE1) | The C-terminally of Mbf1 was tagged with six His epitopes at their natural chromosomal locations in BY4741(*yrr1Δ,* pJFE1) | This study |
| BY4741(*yrr1Δ, TMA17*-6×his, pJFE1) | The C-terminally of Tma17 was tagged with six His epitopes at their natural chromosomal locations in BY4741(*yrr1Δ,* pJFE1) | This study |
| BY4741(*yrr1Δ, HAA1-6*×his, pJFE1-*YRR1*) | BY4741(*yrr1Δ, HAA1-* 6×his, pJFE1) was compensated with *YRR1* | This study |
| BY4741(*yrr1Δ, MBF1*-6×his, pJFE1-*YRR1*) | BY4741(*yrr1Δ, MBF1-*6×his, pJFE1) was compensated with *YRR1* | This study |
| BY4741(*yrr1Δ, TMA17*-6×his, pJFE1-*YRR1*) | BY4741(*yrr1Δ, TMA17-*6×his, pJFE1) was compensated with *YRR1* | This study |
| BY4741(*HAA1*, *TMA17*, *MBF1*) | BY4741 with *HAA1*, *TM17*, *MBF1* overexpression | This study |
| BY4741(*yrr1Δ,* pJFE3) | BY4741 derivate; yrr1::loxP, pJFE3 | This study |

Table S2 List of primers used for plasmids and strain construction in this work

| Primer name | Sequence (5’-3’) |
| --- | --- |
| CRZ1-F | CGCGGATCCATGTCATTCAGCAACGGAAATATGG |
| CRZ1-R | CGACGTCGACTTAACTCTCTTGTCCCGATTTCTCC |
| LSM5-F | CGCGGATCCATGAGTCTACCGGAGATTTTGC |
| LSM5-R | CGACGTCGACTTACAACGCCTCCGTAGGG |
| CGR1-F | CGCGGATCCATGGTGAATGAAACAGGAGAAAG |
| CGR1-R | GGTTCTGCAGCTAACGTTCTTTTAAAGCCTTGTTCC |
| TIF11-F | CGCGGATCCATGGGTAAGAAAAACACTAAAGGT |
| TIF11-R | GGTTCTGCAGTTAAATGTCATCAATATCAAGTTCTTCATCTTC |
| GIS2-F | CGCGGATCCATGTCTCAAAAAGCTTGTTACGT |
| GIS2-R | GGTTCTGCAGCTAAGCCTTTGGACAATCCTTG |
| TAF10-F | CGCGGATCCATGGATTTTGAGGAAGATTACGATG |
| TAF10-R | GGTTCTGCAGCTAACGATAAAAGTCTGGGCGAC |
| TMA17-F | CGCGGATCCATGTGCTCAGCAGGCGG |
| TMA17-R | GGTTCTGCAGTTAAATGAATATGCTGTTTGGTGTCAC |
| HSP12-R | GGTTCTGCAGTTACTTCTTGGTTGGGTCTTCTTCA |
| HSP12-F | CGCGGATCCATGTCTGACGCAGGTAGAAAAG |
| DEF1-F | CGCGGATCCATGTCTACACAATTTAGGAAGTCTAATC |
| DEF1-R | GGTTCTGCAGTTAGTAGAAACCTCTTGAATTTTTAGAATTGT |
| DSK2-F | CGCGGATCCATGTCGTTGAATATACATATCAAGTCAG |
| DSK2-R | CGACGTCGACTTAAACATCGCCGTTCAGTAGT |
| MBF1-F | GCTCTAGAATGTCTGACTGGGATACAAATACTAT |
| MBF1-R | GGTTCTGCAGTCATTTCTTCTTTGGAGCTCCCA |
| HAA1-F | CGCGGATCCATGGTCTTGATAAATGGCATAAAGTATG |
| HAA1-R | GGTTCTGCAGTCATAACGAAGACATGAAATTATCCAAATC |
| HAA1-his-F1 | GAAGTTATTAGGTGATATCAGATCCACTAGTGGCCTATGTAAATTTGCTTTTCGTCTCTTGCTTCTCTG |
| HAA1-his-R1 | CGCACCTAGAAAGGTTGAGAATGC |
| HAA1-his-F2 | GAAGTTATTAGGTGATATCAGATCCACTAGTGGCCTATGTAAATTTGCTTTTCGTCTCTTGCTTCTCTG |
| HAA1- his-R2 | CGCACCTAGAAAGGTTGAGAATGC |
| MBF1-his-F1 | GAAGTACGGATCAACCAATACGAGG |
| MBF1-his-R1 | ATTAAGGGTTGTCGACCTGCAGCGTACGAAGCTTCAGCTGTCAGTGGTGGTGGTGGTGGTGGGGGCCACCACCTTTCTTCTTTGGAGCTCCCAAAGGC |
| MBF1-his-F2 | GAAGTTATTAGGTGATATCAGATCCACTAGTGGCCTATGAGAGGTCTTCTTTCATTTGTTCCTTTC |
| MBF1-his-R2 | CAACCATTACTCGCATACAATGATG |
| TMA17-his-F1 | CCGTCTTGAAGCTGACGACAG |
| TMA17-his-R1 | ATTAAGGGTTGTCGACCTGCAGCGTACGAAGCTTCAGCTGTTAGTGGTGGTGGTGGTGGTGGGAACCACCACCAATGAATATGCTGTTTGGTGTCACGAC |
| TMA17-his-F2 | GAAGTTATTAGGTGATATCAGATCCACTAGTGGCCTATGGAGACGGGATTTAGTTTTACTGCGC |
| TMA17-his-R2 | GTACTCCTTCACCATATAAGCTCTACGT |
| ﻿TMA17-2f | ﻿CGCGGATCCATGTGCTCAGCAGGCG |
| TMA17-2r | ﻿TGACGTCGACTTAAATGAATATGCTGTTTGGTGTCAC |
| MBF1-2f | ﻿ATTTGCGGCCGCATGTCTGACTGGGATACAAATACT |
| MBF1-2r | ﻿CGAGCTCTCATTTCTTCTTTGGAGCTCCC |

**Additional figures**


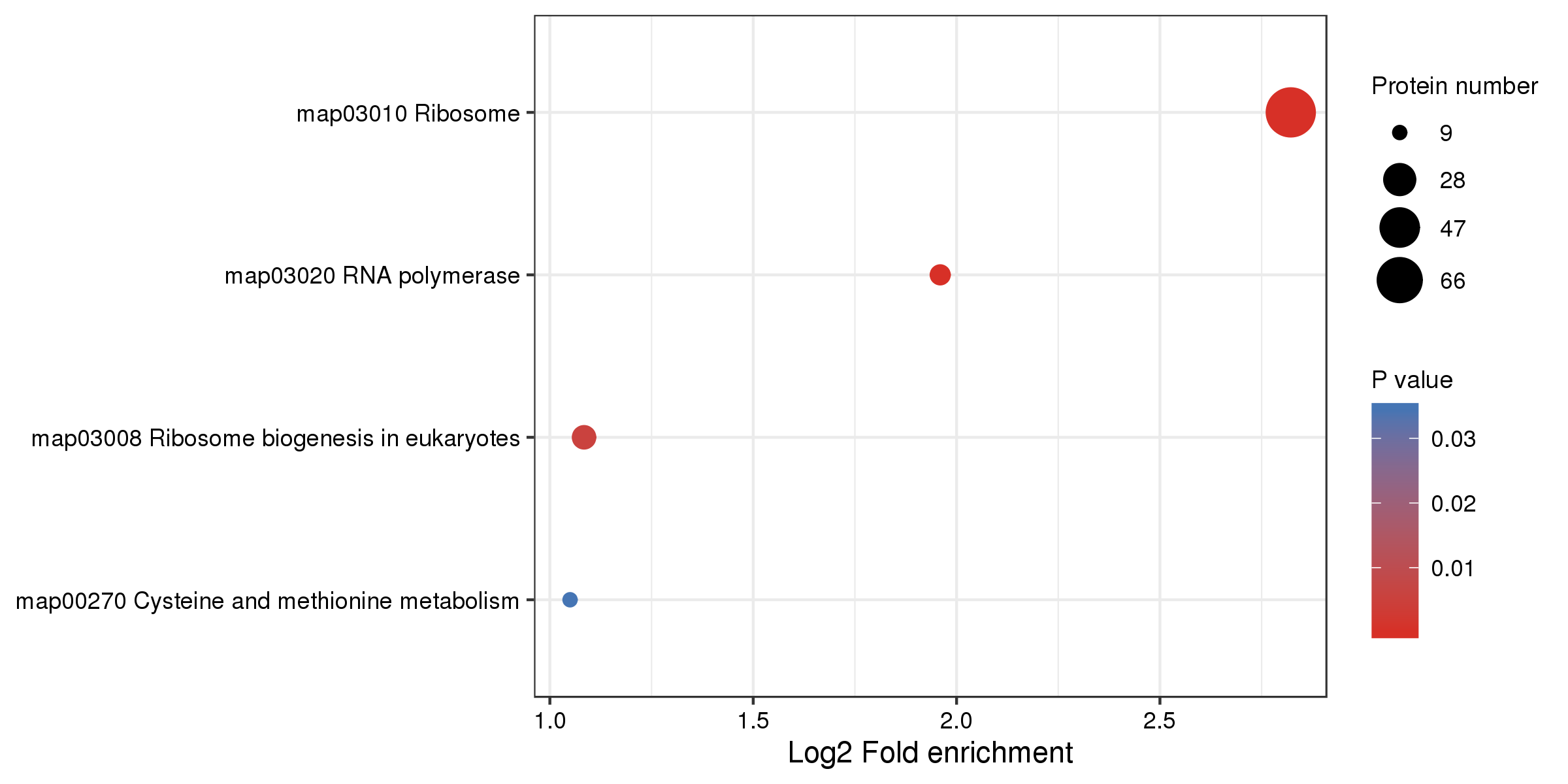


**Fig. S1** Influence of vanillin stress on KEGG pathway enrichment of down-regulated DEPs in CEN.PK2-1C background strains. Greater RichFactor indicates a greater effect of vanillin on the analyzed pathway


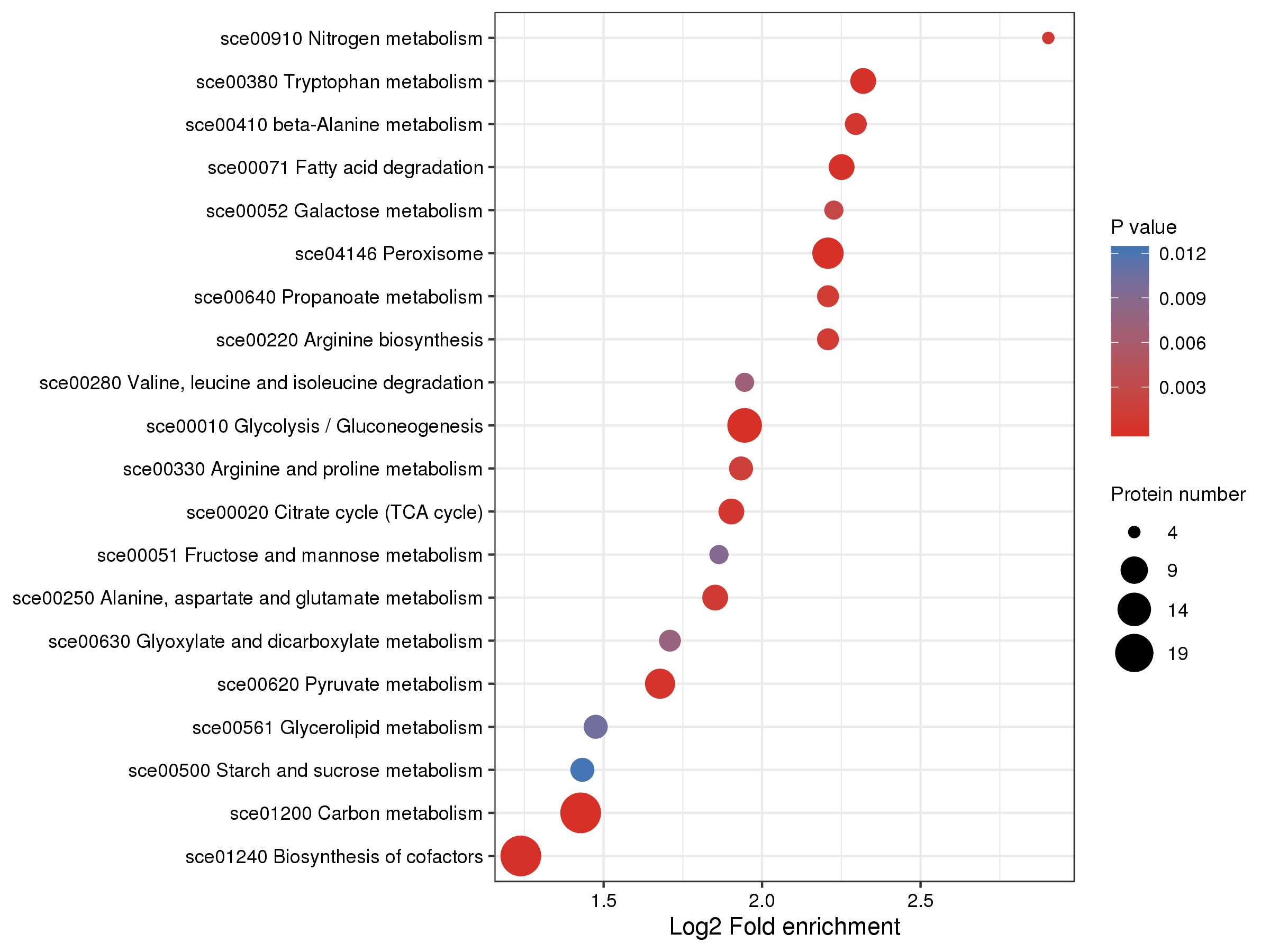


**Fig. S2** Influence of vanillin stress on KEGG pathway enrichment of upregulated DEPs in CEN.PK2-1C background strains. Greater RichFactor indicates a greater effect of vanillin on the analyzed pathway.


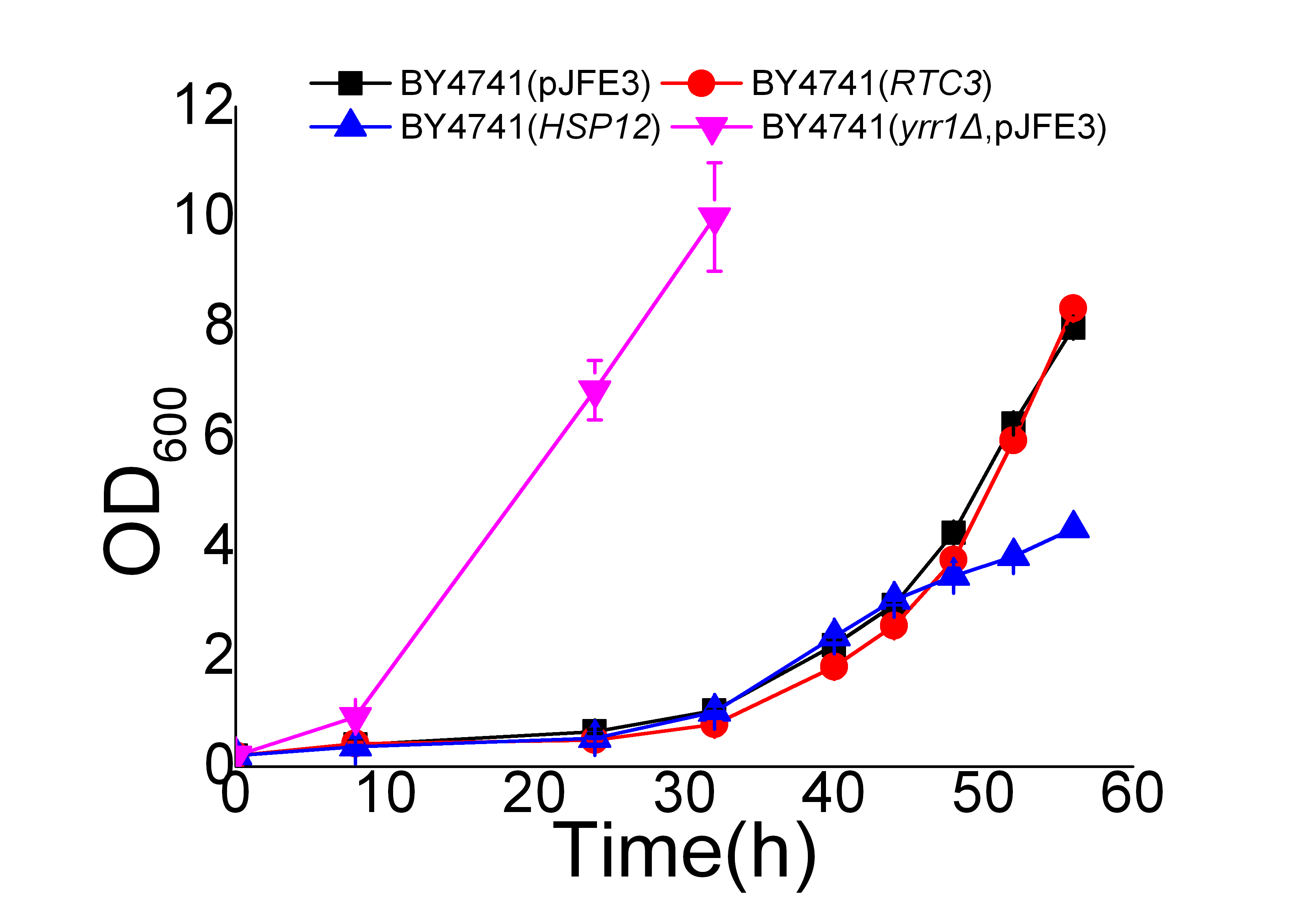


**Fig. S3** Growth curve of overexpression of *RTC3*，*HSP12* in presence of 6 mmol L^-1^ vanillin. BY4741(pJFE3) and BY4741(*yrr1Δ*, pJFE3) are the controls. Data are presented as the means ± standard errors of independent triplicate experiments.

*
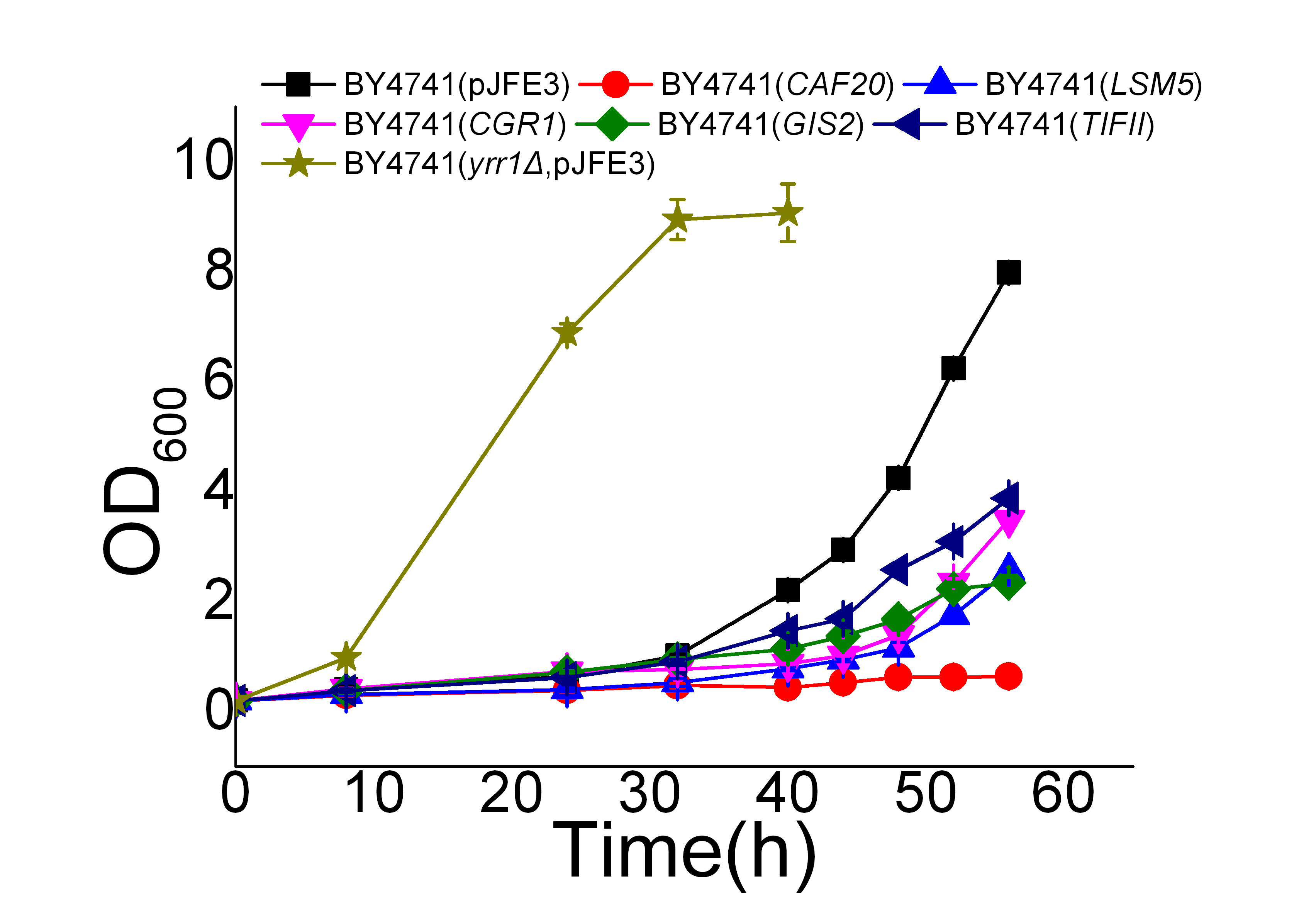
*

**Fig. S4** Growth curve of strains overexpressing translation related proteins encoding genes *CAF20, LSM5*, *CGR1*, *GIS2*, *TIF11*cultured in SC-URA with 6 mmol L^-1^ vanillin. BY4741(pJFE3) and BY4741(*yrr1Δ*, pJFE3) are the controls. Data are presented as the means ± standard errors of independent triplicate experiments.

|  |
| --- |
|  |
|  |

**Fig. S5 Spot growth of BY4741(*yrr1 Δ*) with different inhibitors.**
